# Redox Aspects of Chaperones in Cardiac Function

**DOI:** 10.3389/fphys.2018.00216

**Published:** 2018-03-16

**Authors:** Claudia Penna, Matteo Sorge, Saveria Femminò, Pasquale Pagliaro, Mara Brancaccio

**Affiliations:** ^1^Department of Clinical and Biological Sciences, University of Torino, Torino, Italy; ^2^Department of Molecular Biotechnology and Health Sciences, University of Torino, Torino, Italy

**Keywords:** cardioprotection, ischemia/reperfusion, heat shock proteins, nitrogen reactive species, reactive oxygen species, redox signaling, mitochondria

## Abstract

Molecular chaperones are stress proteins that allow the correct folding or unfolding as well as the assembly or disassembly of macromolecular cellular components. Changes in expression and post-translational modifications of chaperones have been linked to a number of age- and stress-related diseases including cancer, neurodegeneration, and cardiovascular diseases. Redox sensible post-translational modifications, such as S-nitrosylation, glutathionylation and phosphorylation of chaperone proteins have been reported. Redox-dependent regulation of chaperones is likely to be a phenomenon involved in metabolic processes and may represent an adaptive response to several stress conditions, especially within mitochondria, where it impacts cellular bioenergetics. These post-translational modifications might underlie the mechanisms leading to cardioprotection by conditioning maneuvers as well as to ischemia/reperfusion injury. In this review, we discuss this topic and focus on two important aspects of redox-regulated chaperones, namely redox regulation of mitochondrial chaperone function and cardiac protection against ischemia/reperfusion injury.

## Introduction

Chaperones are proteins responsible for folding, transport, maturation, assembly, and activation of many different proteins, impacting on a wide variety of cellular processes. Chaperones act by binding to protein unfolded domains and, through different mechanisms, promoting protein re-folding and/or avoiding the formation of toxic misfolded protein aggregates. Moreover, they promote the degradation of proteins irreversibly misfolded via the ubiquitin-proteasome pathway or through autophagy. Chaperones play an important role in preserving proteins that exert their functions through considerable conformation changes, like receptors and signal transduction mediators. In these cases, protein activation and de-activation imply cyclic structural reorganization, potentially dangerous for protein stability. Moreover, chaperones play an important role in the assembly of multiprotein complexes, likely by facilitating the structural changes caused by the association of the different subunits. Chaperone proteins function in different cell compartments, including endoplasmic reticulum (ER) and mitochondria. In the ER, a specific set of chaperones is devoted to assisting the folding of proteins during their maturation, but they also take part to a quality control machinery, that induces degradation of proteins that fail to reach their active conformation and in generating signals that increase chaperone transcription and translation in case of massive protein unfolding (the Unfolded Protein Response; Groenendyk et al., 2010). In mitochondria maintaining protein folding is particularly challenging due to the specific mechanism of protein import, the presence of reactive oxygen species (ROS) and the need to assemble proteins synthesized in the cytoplasm and inside the organelle in large multiprotein complexes. Mitochondria possess specific chaperones able to face these difficult tasks and to perform a dedicated quality control (Haynes and Ron, 2010). Furthermore, chaperones are even secreted by cells and can act in the extracellular milieu by chaperoning secreted factors or by signaling through membrane receptors (Eustace and Jay, 2004; Calderwood et al., 2016).

In summary, chaperone proteins exert a number of essential functions in eukaryotic cells in physiological situations, although their action is even more crucial under stress conditions. In this review, we will focus on the role of chaperone proteins in the heart. Indeed, cardiomyocyte cytoplasm is crowded with proteins, mainly forming the specialized contractile apparatus, that interact the ones with the others building solid complexes, able to cope with relevant mechanical stress. The proper folding, assembly and turnover of this multitude of proteins depend on chaperone protein activity (Christians et al., 2014; Tarone and Brancaccio, 2014). Protein misfolding can occur due to a number of means, as genetic mutations, inaccurate post-translational modifications, excessive mechanical stretch and ROS production. Notably, the accumulation of misfolded proteins characterizes a number of cardiac diseases, like hypertrophic cardiomyopathy, idiopathic dilated cardiomyopathy, myocardial infarction and genetic cardiomyopathies. Furthermore, the induction of protein aggregates causes cardiomyopathy in animal models (Bulteau et al., 2001; Tannous et al., 2008; Tian et al., 2012; Parry et al., 2015). Accordingly, the forced expression of chaperone proteins generally protects the heart from many different stress conditions, including the production of ROS (Tarone and Brancaccio, 2014).

In this review, we consider the response of chaperones to ROS production, the redox aspects that can influence chaperone function within the heart during ischemia/reperfusion and chaperone importance in protecting the heart from deleterious consequences. We also focus on chaperone role within mitochondria, as these organelles are extremely important in cardiac activity both in physiologic and pathological conditions.

## Reactive oxygen and nitrogen species and redox signaling

ROS derive from several enzymatic activities in cardiomyocytes and other cells of the cardiovascular system (e.g., endothelial and smooth muscle cells), with mitochondria representing the main source for their production. Indeed, about 2% of oxygen (O_2_) consumed by cardiac mitochondria is transformed to superoxide anion (${\text{O}}_{2}^{–•}$) due to the incomplete reduction of O_2_. Therefore, ${\text{O}}_{2}^{–•}$ is mainly a byproduct of aerobic respiration. Following spontaneous dismutation, it is transformed to hydrogen peroxide (H_2_O_2_), which via the Fenton reaction can be converted in the highly reactive hydroxyl radical (OH^•^) (Turrens, 2003; Tullio et al., 2013; Henstridge et al., 2016). H_2_O_2_ can be inactivated by glutathione catalyzed by glutathione peroxidase or catalase (Radi et al., 1991; Arai et al., 1999).

Reactive nitrogen species (RNS) refer to the reactive molecules stemming from nitric oxide (NO^•^), the signaling gaseous molecule, which in certain conditions may act as an antioxidant. It is mainly synthesized from L-arginine by the nitric oxide synthases, NOS1 (neuronal NOS, nNOS), NOS2 (inducible NOS, iNOS) and NOS3 (endothelial NOS, eNOS). In the heart, NOS3 is mainly found in the caveolae of coronary vascular endothelium, whereas cardiomyocytes constitutively express NOS1 and NOS3 in different subcellular structures, while inducible NOS2 expression can be triggered by several stimuli, including infections, heart failure and ischemia/reperfusion (Brown and Borutaite, 2007; Tullio et al., 2013; Penna et al., 2014a). The possibility that mitochondria are important sources of NO^•^ via a mitochondrial NOS (mitoNOS) variant has been proposed. However, definitive evidence concerning the existence of mitoNOS is not yet available (Lacza et al., 2009).

Nitric oxide can be formed also by other enzymatic and non-enzymatic reactions (Penna et al., 2014a). It can also be transformed, by a redox reaction, into many reactive molecules including nitroxyl (HNO), nitrite (${\text{NO}}_{2}^{-}$), and peroxynitrite (ONOO^−^), each one has different functional effects (Wink et al., 2003; Brown and Borutaite, 2007; Tocchetti et al., 2011; Tullio et al., 2013; Penna et al., 2014a).

Under pathophysiological conditions, excessive, unbalanced, ROS and RNS formation may be deleterious for organelle and cell activity as they can accumulate and damage proteins, lipids, and DNA. However, it is now clear that they are also involved in many important signaling functions. Moreover, ROS and RNS can interact to shift from a form to another with more or less reactivity potential. ROS/RNS can induce discrete, reversible and site-specific modifications in proteins, controlling a redox signaling in physiological changes in channel and enzyme function, as well as regulation of transcription. On the other hand, ROS/RNS can induce alterations, diffuse and irreversible, defined as redox stress. The latter is involved in pathophysiological processes together with other pathologic conditions such as inflammation. Proteins more commonly targeted by ROS/RNS contain sensible amino acidic side chains, including cysteine, methionine and histidine, or coordinated metal centers that regulate their conformation and function (Giles et al., 2003). For instance, NO^•^ can react with ${\text{O}}_{2}^{–•}$ to form ONOO^−^, which can impair directly and irreversibly tyrosines in the proteins, yielding tyrosine nitration, a result that is frequently deleterious. NO^•^ can react also with ONOO^−^ to form N_2_O_3_ which can, in turn, react with the so-called “reactive cysteines” to yield S-nitrosylation or S-nitrosylated proteins (PSNO), a result that is frequently beneficial. Indeed, S-nitrosylation sheds cysteine from further oxidation processes.

Cysteine residues of proteins display chemistry versatility, thus these residues may achieve multiple oxidation states and, therefore, are particularly suited to the task of switching from signaling to stress. Indeed, the reactive cysteines can react with ROS/RNS to produce a number of species comprising PSNO. Reactive cysteines can also react with glutathione to form mixed disulfides, such as S-glutathionylation (see below) (Hurd et al., 2005a; Penna et al., 2014a). Intra- and inter-molecular disulfide bonds (PSSP) can also occur. If ROS are excessive, protein thiol oxidation can progress to sulfinic acid (PSO_2_H) and sulfonic acid (PSO_3_H), leading to irreversible protein dysfunction, representing redox stress (Hurd et al., 2005b).

S-nitrosylation, which is emerging as the paradigm of redox signaling, is the incorporation of nitric oxide moiety to a sulfur atom to form the SNO bond in proteins (PSNO). S-nitrosylation being a signaling modality that acts as a reversible molecular switch resembles the phosphorylation/dephosphorylation in kinase signaling (Hess et al., 2005; Penna et al., 2014a).

An interesting process is the formation of a disulfide bond (PSSP) with a concomitant release of NO-moiety that take place by nucleophilic attack of proximal protein thiols to the site of SNO. While S-nitrosylation may occur with different reaction mechanisms, denitrosylation may be due to S-nitrosoglutathione reductase and/or the intervention of thioredoxin system (namely the cytosolic Trx1 and the mitochondrial Trx2). These are considered the two main enzymatic systems for denitrosylation, which, actually, may also occur for non-enzymatic processes (Sengupta et al., 2007; Benhar et al., 2008; Penna et al., 2014a). For instance, thioredoxins denitrosylate proteins and then thioredoxin reductase regenerates thioredoxins (Penna et al., 2014a).

As said, another modification of protein cysteine residues representing a redox signaling modality is S-glutathionylation, which refers to a covalent modification of a cysteine residue by glutathione. S-glutathionylation comprises a mixed disulfide species (PS-SG). Proteins particularly rich in cysteine are susceptible to glutathionylation especially in the presence of alkaline pH, which favors deprotonation of protein thiols and reaction with glutathione. Of course, S-glutathionylation, like other signaling processes, is reversible through a process called deglutathionylation. In this process, the main enzymes involved are glutaredoxin and thioredoxins. Also, reduction of disulfides may be responsible for deglutathionylation when the GSH pool is reduced (Beer et al., 2004).

S-nitrosylation and S-glutathionylation interact: glutathione reductase removes the NO fraction from proteins through the transnitrosation of SNO with GSH to form GSNO (S-nitrosoglutathione), which will, in turn, be converted into GSH by S-nitrosoglutathione reductase.

**Figure 1:**
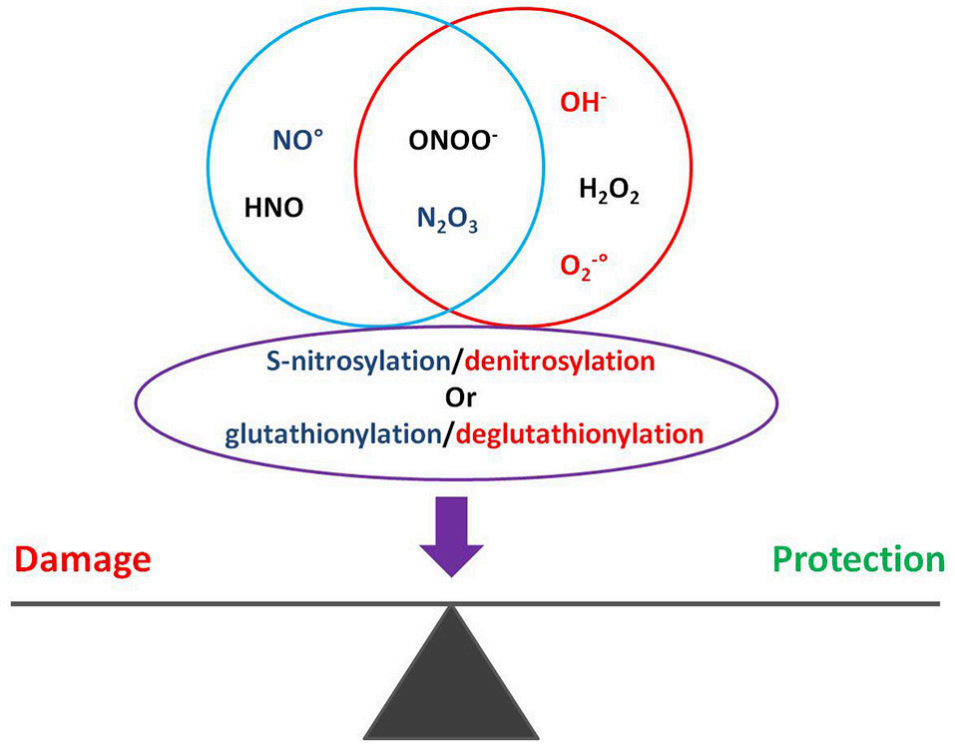
Oxygen and nitrogen reactive species regulate S-nitrosylation and glutathionylation of proteins, impacting on cardiac pathophysiology.

The dynamic S-nitrosylation/denitrosylation and glutathionylation/deglutathionylation reactions are of pivotal importance in the regulation of the cardiovascular system. For example, in transgenic models of increased or decreased activity of S-nitrosoglutathione reductase, the sepsis-induced myocardial depression is positively influenced by denitrosylation/deglutathionylation. Therefore, besides guanylyl cyclase activation and cGMP production, NO^•^ may affect pathophysiology via S-nitrosylation and S-glutathionylation of proteins, including chaperones (Figure 1; Sengupta et al., 2007; Benhar et al., 2009; Anand and Stamler, 2012; Beigi et al., 2012; Martínez-Ruiz et al., 2013).

As described above and as reported below, cells have evolved multiple fine-regulated systems to balance their redox homeostasis, including detoxifying enzymes and reducing proteins. The regulation of ROS levels is a critical point in cells. Indeed, ROS/RNS play important roles in different signaling pathways (Rhee, 2006; D'Autreaux and Toledano, 2007; Tullio et al., 2013). However, once ROS exceed the antioxidant capacity of the cell, it causes oxidative stress. ROS can induce important alterations in the function and structure of DNA, lipids and in particular proteins rich in cysteine, methionine, and histidine (Imlay, 2003). Interestingly, these ROS-sensible amino acidic side chains are frequently present in chaperone proteins, suggesting that ROS can regulate chaperone oxidation state and activity under oxidative stress conditions.

## Redox regulation of chaperone proteins

Chaperones are highly conserved proteins that may play a huge protective role during cellular stress and pathologic conditions. Important redox modifications of chaperone proteins have been described in oxidative stress conditions.

HSP33 is a well-known redox-activated chaperone protein in prokaryotes that protects bacteria from oxidative stress damage, preventing protein unfolding and aggregation (Winter et al., 2008). *In vitro* experiments demonstrated that HSP33 is activated by hydrogen peroxide and heat shock through the formation of reversible disulfide bonds. A first disulfide bond in the C-terminal domain of the protein induces the unfolding of its zinc-binding domain and the linker region between the C-terminal and the N-terminal substrate-binding domain. The formation of a second disulfide bond blocks the linker region in the unfolded conformation, exposing a highly hydrophobic surface. This favors the interaction between two oxidized HSP33 in a stable chaperone-active homodimer that binds unfolded proteins under oxidizing condition (Graumann et al., 2001; Ilbert et al., 2007).

Asna1/TRC40 (transmembrane recognition complex 40), similarly to HSP33, functions as a redox-regulated chaperone during oxidative stress, in eukaryotes (Voth and Jakob, 2017). In non-stress conditions, Asna1/TRC40 is devoted to facilitating the post-translational delivery of tail-anchored proteins to the mammalian endoplasmic reticulum. However, in oxidative conditions, the formation of a disulfide bond induces a conformational change in the protein that oligomerizes and behaves as a molecular chaperone. The possibility that Asna1/TRC40 protects mammalian tissues from oxidative stress is still under investigation (Voth and Jakob, 2017). Moreover, also other proteins as the PLP-dependent aminotransferases hBCAT or the antioxidant enzymes 2-cys peroxiredoxins are known to be oxidized under severe oxidative stress condition, inducing the formation of high molecular weight oligomers or supra-molecular complexes with chaperone activity (Jang et al., 2004; El Hindy et al., 2014). In eukaryotes, redox alterations are described in various chaperone proteins, as GRP58, GRP78, HSC70, HSP90, HSP70, and HSP60 (Fratelli et al., 2002; Lind et al., 2002; Scroggins and Neckers, 2007; Wang et al., 2012). HSP90 is one of the most important molecular chaperones, involved in different signaling pathways in normal and pathological conditions. HSP90 activity is determined by its ability to hydrolyze ATP and is regulated by the binding with small co-chaperone proteins. Intriguingly, the HSP90 function is modulated also by various post-translational modifications, as phosphorylation, acetylation, ubiquitination, S-nitrosylation and oxidation (Scroggins and Neckers, 2007). In endothelial cells, NO^•^ binds to the thiol side chain of cysteine-597 in the C-terminal domain of HSP90, compromising its ATPase activity and thus inhibiting its chaperone function (Martínez-Ruiz et al., 2005). In human breast cancer MDA-MB-231 cells, the treatment with the cytotoxic steroid tubocapsenolide A induces an increase in oxygen reactive species and a reduction in intracellular glutathione content. In this condition, ROS determine a thiol oxidation of HSP90 causing the loss of its chaperone activity and the consequent proteasomal degradation of its client proteins (Chen et al., 2008).

TRAP1 (tumor necrosis factor receptor-associated protein 1), the mitochondrial homolog of HSP90, is subjected to S-nitrosylation in tumor cells lacking S-nitrosoglutathione reductase (GSNOR). S-nitrosylation at cysteine-501 causes an accelerated proteasomal degradation of TRAP1, inducing an increase in levels and activity of succinate dehydrogenase (SDH), normally inhibited by TRAP1, sensitizing cells to SDH-inhibitors chemotherapy. Other aspects of TRAP1 function are described below (see “Chaperones in mitochondria”).

Protein Disulfide-Isomerase (PDI) is another well-known redox-dependent chaperone in humans, ubiquitously expressed and mainly localized in the endoplasmic reticulum (Hatahet and Ruddock, 2009). PDI catalyzes the folding of its substrates under oxidative conditions. PDI activity is related to its conformation, which is in turn dependent on the redox state of its active sites. In particular, the oxidation of its active sites determines the conversion of PDI from a compact to an open conformation exposing the substrates-binding surface. PDI binds to unfolded proteins and, by reducing its own disulfide bonds, induces the formation of disulfides in the substrate. The reduction of its active sites determines the return to its compact conformation, releasing the folded substrate (Wang et al., 2015).

HSP27 is a molecular chaperone active when aggregated in a high molecular weight complex. In ischemia/reperfusion (I/R) injury, HSP27 cysteine-141 forms a disulfide with a low-molecular-weight thiol, such as glutathione (S-glutathionylation), and this modification induces the disaggregation of the multimeric complex and the loss of its chaperone activity (Eaton et al., 2002).

HSP70 and HSP60 are two other chaperones susceptible to S-glutathionylation under oxidative stress conditions. The overexpression of HSP70 is protective against oxidative damage in H9C2 cells exposed to oxidative reagents or hypoxia as well as *in vivo* models subjected to ischemic injury (Marber et al., 1995; Chong et al., 1998; Okubo et al., 2001). It has been suggested that S-glutathionylation of HSP70 may potentiate its chaperone activity (Fratelli et al., 2002). A similar regulation is proposed also for HSP60, a predominantly mitochondrial chaperone known to be upregulated by the accumulation of unfolded and oxidized molecules within mitochondria. In accordance, HSP60 overexpression protects against ischemia-reperfusion injury (Fratelli et al., 2002; Lind et al., 2002).

## Chaperones in the heart

The intense, restless contractile and metabolic activities of the heart require a continuous supply of oxygen and nutrients and a tight control of synthesis, folding and turnover of macromolecules, including proteins. In the case of cardiac overload and in stress conditions, the greater myocardial energy demand may enhance the leakage of electrons from mitochondrial complexes I to III and may induce ROS formation, which may overcome the anti-oxidant cell capacity, thus damaging proteins and other target molecules. Indeed, the accurate regulation of cardiac proteostasis may be impaired by several stress conditions, including oxidative stress, causing an accumulation of damaged and misfolded proteins that exceed the cellular degradation ability. Unfolded proteins can thus aggregate in toxic oligomers and finally in bigger insoluble aggregates disrupting cardiomyocyte structure and function and leading to cardiomyopathy (Willis and Patterson, 2013; Del Monte and Agnetti, 2014; McLendon and Robbins, 2015). Nevertheless, oxidative stimuli may also induce the activation of intracellular signaling pathways to sustain the cardiac activity and prevent the onset of cardiomyopathy (Tarone and Lembo, 2003; Sorge and Brancaccio, 2016). Of note, proteins involved in signal transduction are often metastable, changing conformation during activation/deactivation processes. This feature confers to these proteins a particular propensity to denaturate in stress conditions (Conway and Lee, 2015).

The heart is rich in chaperones and co-chaperones and under stress conditions, it increases chaperone expression and activity to cope with unfolded protein accumulation and sustain the activation of protective pathways. Indeed, in the heart small heat shock proteins and bigger chaperones, as HSP90 and HSP70, work in a coordinated fashion to regulate the intracellular signaling cascade and the folding or degradation of unfolded proteins (Hartl et al., 2011; Kriegenburg et al., 2012; Tarone and Brancaccio, 2014).

HSP90 and HSP70 are considered the two most important molecular chaperones and are both rapidly induced in stressed hearts. The interaction with co-chaperones, that regulate the binding to specific targets, allow them to induce the conformational changes needed to activate/deactivate signaling molecules and their assembly in pro-survival signalosome complexes (Kupatt et al., 2004; Tarone and Brancaccio, 2014; Parry et al., 2015). This chaperone machinery also promotes the correct folding of specific “clients proteins” (http://www.picard.ch/downloads/downloads.htm) and controls the degradation of unfolded proteins and of protein aggregates, through the proteasome and the autophagosome pathways (Ficker et al., 2003; McDonough and Patterson, 2003; Carra et al., 2008; Arndt et al., 2010; Taipale et al., 2010).

Small heat shock proteins are another group of chaperones characterized by a α-crystallin domain which favors their oligomerization in supramolecular complexes with chaperone activity. Recent experimental evidence suggests an important role for these chaperones in the stressed heart. Indeed, by binding their client proteins, in some cases in association with HSP90, they are able to promote protein folding, prevent misfolded protein aggregation and support signal transduction pathways (Vos et al., 2011; Bakthisaran et al., 2015; Haslbeck and Vierling, 2015).

For example, αB-crystallin (CryAB or HSPB5) is a small chaperone fundamental for cytoskeletal proteins folding, in particular for desmin and titin. A missense mutation in its coding gene induces the formation of desmin aggregates and causes a cardiomyopathy (Vicart et al., 1998).

Other small heat shock proteins, like HSP27, HSP20, HSP22, and Melusin are induced under stress conditions favoring protein folding and pro-survival signaling activation (Sui et al., 2009; Fan and Kranias, 2011; Christians et al., 2012; Sorge and Brancaccio, 2016). The mitochondrial and protective role of these and other chaperones are described in the following paragraphs.

## Chaperones in cardiac mitochondria

**Figure 2:**
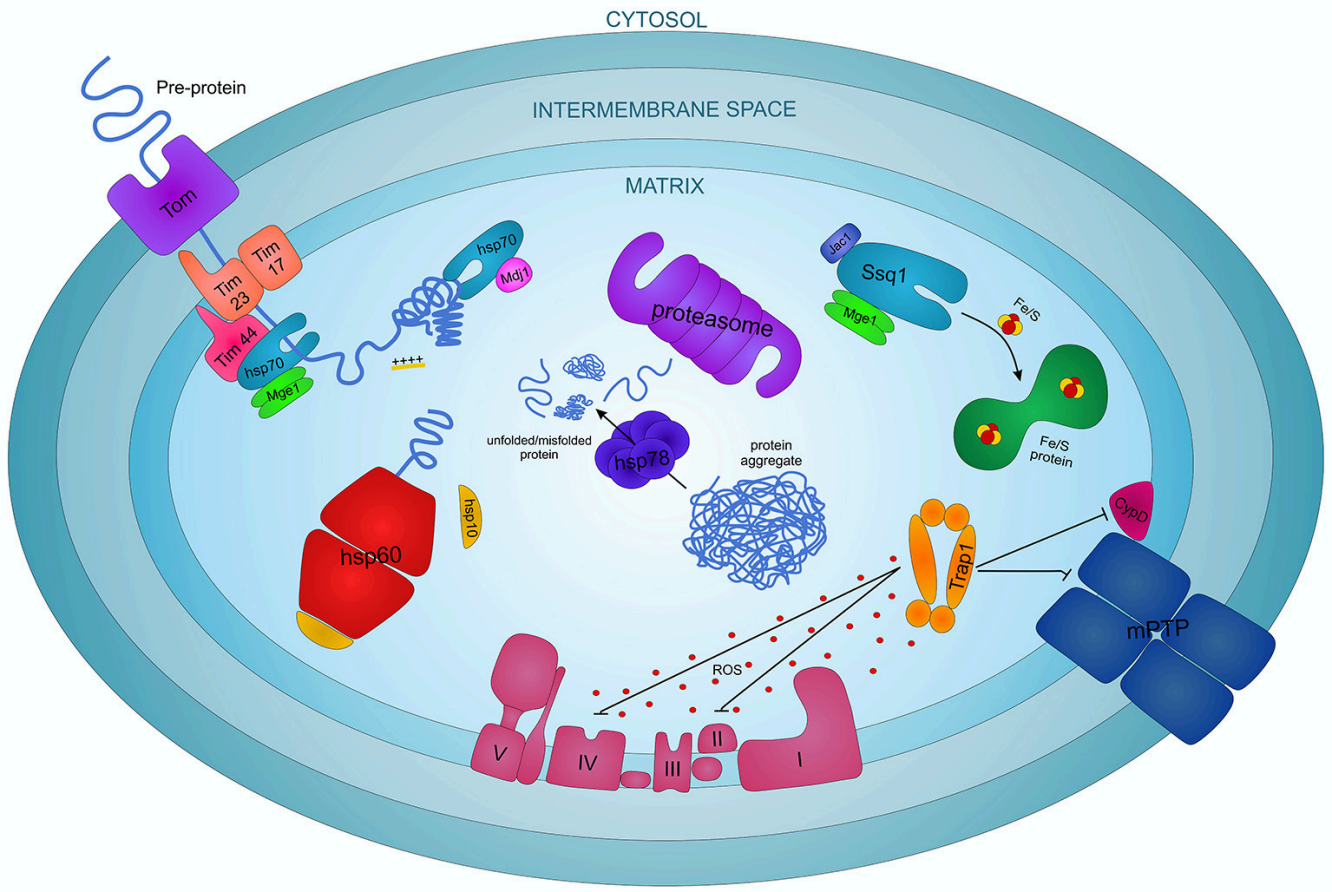
The complex interplay of chaperone proteins in regulating the apoptotic process in cardiomyocytes.

Mitochondria represent 35–40% of cardiac cell volume. Therefore the role of chaperones in these organelles is very important for cardiac function, especially in I/R and cardioprotection. Mitochondrial chaperones have been extensively studied in Saccharomyces cerevisiae elucidating a complex system that can be generalized to all the eukaryotes. In these organelles, specific chaperones assist proteins in their import and folding and protect mitochondria from different stress stimuli, like temperature and excessive ROS (Figure 2).

Mitochondrial HSP70 (mtHSP70), also known as Ssc1, Grp75, PBP74, mot-2 or mortalin, is considered the most important mitochondrial chaperone in higher eukaryotes. It is a member of the HSP70 family characterized by an N-terminal ATPase domain and a C-terminal peptide-binding domain. mtHSP70 is translated into the cytoplasm and transported into mitochondria where it interacts with structural mitochondrial proteins, metabolic enzymes and proteins involved in cell differentiation and survival (Wadhwa et al., 1998, 2002, 2003; Schwarzer et al., 2002). mtHSP70 is essential for pre-protein—precursors of mature proteins—import from cytosol to the mitochondrial matrix. Pre-proteins cross the double mitochondrial membrane in an extended conformation thanks to a positive charged N-terminal part, favored by the membrane electrochemical gradient (Schwartz et al., 1999). mtHSP70 forms a complex with its co-chaperone Mge1, a homolog of the bacterial Grp3 protein, that induces the release of ADP and P_i_ augmenting the ATPase activity of mtHSP70 (Dekker and Pfanner, 1997). Mge1 may stabilize the interaction of mtHSP70 with the inner membrane protein Tim44 to create a molecular motor that uses the energy from the ATP hydrolysis for the full translocation of the polypeptide chains inside the mitochondria (Figure 2; Wachter et al., 1994; Schneider et al., 1996).

Another important role of mtHSP70, not related to the translocase activity, is in protein folding and prevention of protein aggregation. Indeed mtHSP70 interacts with another cochaperone, Mdj1, a homolog of the bacterial DnaJ protein, and they associate with newly imported or neo-synthesized proteins or with misfolded aggregate proteins to mediate their folding to the native conformation (Figure 2; Herrmann et al., 1994; Prip-Buus et al., 1996).

Ecm10 and Ssq1 are two other HSP70 family proteins contained within mitochondria. Ecm10 is a very close homolog of mtHSP70, likely involved in different functions (Baumann et al., 2000). Ssq1 is a mtHSP70 homolog involved in the maintenance of the mitochondrial genome and in the assembly of iron/sulfur (Fe/S) containing complexes (Schilke et al., 1996, 1999). In particular, Ssq1, regulated by the co-chaperone proteins Mge1 and Jac1, is responsible for the formation of Fe/S clusters and for their assembly into functional protein complexes (Figure 2; Lutz et al., 2001; Schmidt et al., 2001).

HSP60 and HSP10, two chaperone proteins translated in the cytoplasm and then imported into the mitochondrial matrix play an important function in mitochondrial protein folding. HSP60 consists of a double ring system each composed of seven protein subunits (Xu et al., 1997). The co-chaperone HSP10, a homolog of the bacterial GroES, forms a cap closing the opening of the inner cavity of the HSP60 double ring, regulating substrate accessibility and ATPase activity (Martin et al., 1993; Fenton et al., 1996; David et al., 2013). Pre-folding proteins and newly imported pre-proteins enter the cavity of the complex and acquire their native conformation thanks to an ATP-dependent reaction (Figure 2; Ostermann et al., 1989; Brinker et al., 2001). Recent studies also indicate that HSP10 can be involved in the RasGTPase pathway, protecting myocytes from I/R damage, and that it interacts with caspase 3 and modulates Bcl-2 family factors, suggesting a potential anti-apoptotic role in cardiomyocytes (Shan et al., 2003; Lin et al., 2004).

HSP78 and Prohibitins are other significant chaperones and proteins involved in the folding process inside the mitochondria. HSP78, activated by heat stress, and Mcx1, members of the HSP100 family mediate protein folding and degradation of irreversibly damaged proteins (Figure 2). Prohibitins, induced after heat shock, oxidative and metabolic stress, act as foldase-unfoldase and control the AAA-proteases activity in protein degradation. Prohibitin complexes control cell proliferation, cristae morphogenesis and the functional integrity of mitochondria (van Dyck et al., 1998; Steglich et al., 1999; Krzewska et al., 2001; Nijtmans et al., 2002).

As said, mitochondria are the main sources of ROS production in the cardiomyocytes and consequently the more subjected to oxidative stress. TRAP1 is induced upon oxidative stress and characterized by an anti-oxidant and anti-apoptotic activity (Montesano Gesualdi et al., 2007). TRAP1 is strictly similar to the cytosolic HSP90, with the same domain organization. However, it has a different mechanism of folding, due to a characteristic asymmetric conformation when bound to the client in its ATP-binding state. The hydrolysis of a first ATP determines a rearrangement of the chaperone conformation and the client folding, then a second ATP hydrolysis induces the release of the client and a switch of TRAP1 conformation to its ADP-binding state (Lavery et al., 2014). TRAP1 has an important role in mitochondrial homeostasis, maintaining organelle integrity and preventing energy depletion under ROS-induced stress. In particular, TRAP1 inhibits the cytochrome oxidase (complex IV of the respiratory chain) and the succinate dehydrogenase (complex II of the respiratory chain and component of the TCA cycle), favoring the metabolic switch from oxidative phosphorylation toward aerobic glycolysis, avoiding ATP depletion and mitophagy under oxidative conditions (Figure 2; Sciacovelli et al., 2013; Yoshida et al., 2013). TRAP1 has also an anti-apoptotic role as it antagonizes the mitochondrial permeability transition pore (mPTP) opening by inhibiting the complex II-induced ROS release and the mPTP inducer Cyclophilin-D (Figure 2; Kang et al., 2007; Guzzo et al., 2014).

New chaperones have been discovered in the last years with multiple functions, as the small Tims chaperones, involved into the import and translocation of proteins and metabolites in the mitochondria, Tid1, a co-chaperone involved in mitochondrial homeostasis and cell apoptosis, and Hep1 that favors mtHSP70 folding and regulate mitochondrial proteostasis (Petrakis et al., 2009; Blamowska et al., 2012; Cheng et al., 2016). It is likely that new mitochondrial chaperones are still to be discovered.

## Chaperones in physiological exercise

It has been demonstrated in animals and humans that physiological stress induced by exercise modulates the activity and expression of HSPs in many tissues. Indeed, during exhaustive or very intense exercise, the temperature of the muscles can reach 45°C, which can represent a thermal shock able to induce HSPs expression. Moreover, other events, such as oxidative stress (i.e., augmented levels of ${\text{O}}_{2}^{•-}$ and H_2_O_2_), muscle damage and inflammatory response, can occur in this type of exercise and may represent stimuli able to induce HSP expression. Nevertheless, although the production of ROS may play an important role in mediating the expression of chaperones, no definitive evidences for a redox mechanism exist in the exercise-induced chaperone expression (Dimauro et al., 2016; Henstridge et al., 2016). Both acute and chronic exercise modulates the expression of specific HSPs in different organs in a sex-dependent and species-specific manner (Dimauro et al., 2016; Henstridge et al., 2016). Several scientific studies have described the probable relationship between induction of different HSPs and ROS generated after acute exercise in humans. Salo et al. (1991) reported that in rats, after intense and exhaustive exercise, the levels of more than 15 HSPs, including HSP70, increase in heart, liver and skeletal muscle tissues. In addition to HSP70, also αB-crystallin, HSP27, HSP60 and HSP90 were described among the chaperones induced by acute exercise in *vastus lateralis* muscle and blood cells (Fehrenbach et al., 2000; Khassaf et al., 2001; Fischer et al., 2006; Petersen et al., 2012). Other studies have suggested that also various types of chronic exercise could promote an adaptive homeostatic process that modulates the expression of different HSPs in humans. Also in this case, HSP70, αB-crystallin, HSP27, and HSP60 are upregulated and associated to a redox response. Indeed, it has been reported a good correlation between oxidative enzymes and HSP expression in skeletal muscle (Vogt et al., 2001; Morton et al., 2008; Cumming et al., 2014) and/or blood cells (i.e., leukocytes) (Simar et al., 2012; Ziemann et al., 2013; Beltran Valls et al., 2014). During repeated bouts of exercise, in the training period, the expression of stress-proteins, as the aforementioned HSPs, can occur together with the expression of antioxidants, leading to a homeostatic adaptation. This adaptation brings gradually back the HSP levels. Indeed, after weeks of training the levels of HSP70 and HSP27 return to pre-exercise levels and animals that have successfully completed a resistance training program no longer show increases in HSPs performing the training exercises. It seems that the acquired pro-reducing conditions of trained animals prevent HSP induction by ROS (Salo et al., 1991; Beltran Valls et al., 2014). Obviously, this will only be true as long as physical exercise is maintained, so that, in case of detraining, it will bring back the redox status and the new exercise bouts will be able to re-induce “stress” and the expression of HSPs (Davies, 2016).

Though the precise mechanisms linking redox aspects and HSP modulation during physiologic exercise is still not completely understood, a ROS-mediated modulation of HSP expression may be present during acute exercise and a putative homeostatic process, underpinning the involvement of several small HSPs, is described for chronic exercise (Cumming et al., 2014).

## Chaperones in myocardial ischemia/reperfusion injury and cardioprotection

In the heart, the mismatch between oxygen supply and demand leads to myocardial ischemia, which has deleterious effects, ranging from contractile impairment to cell death. The damage due to ischemia may be exacerbated by reperfusion, inducing an I/R injury.

ROS/RNS formation and impaired antioxidant capacity are among the proposed mechanisms to explain the myocardial I/R injury. The unbalanced redox changes lead to the dysfunction of protective molecules against cellular death, including deregulation of chaperones and co-chaperones. Indeed, if chaperones do not work properly, misfolded proteins cannot be repaired and may form insoluble aggregates. These aggregates are highly dangerous for the cells and may participate in the processes leading to cardiomyocyte death and consequently to cardiovascular diseases, such as arrhythmias, dilated cardiomyopathy and heart failure (Willis and Patterson, 2010, 2013; Tarone and Brancaccio, 2014, 2015).

Although prolonged episodes of ischemia followed by reperfusion induce damage, transient episodes (a few minutes or seconds) of ischemia before or after a prolonged cardiac ischemia may induce cardioprotection with consequent reduction of infarct size, myocardial dysfunction, and arrhythmias. These procedures are referred to as ischemic preconditioning (IP) or post-conditioning (PostC), respectively (Penna et al., 2014a). Also, transient episodes of ischemia in a remote organ before (remote ischemic pre-conditioning), during (remote per-conditioning) or after (remote post-conditioning) a prolonged ischemic insult can attenuate myocardial I/R injury (Lau et al., 2017). Other physiological procedures able to induce cardioprotection are repeated short-term episodes of exercise (exercise preconditioning), that can trigger a phenotype similar to that induced by IP (Yuan et al., 2018). The same protection could be obtained with pharmacological tools (Penna et al., 2014a), given before, during or after an ischemic insult, known as pharmacological conditioning. The cardioprotective mechanisms of the various conditioning procedures (ischemic, remote or pharmacological) are strongly associated. They may induce two windows of cardioprotection: early preconditioning (first window of protection) and late preconditioning (second window of protection) (Yuan et al., 2018).

Ischemia/reperfusion as well as cardioprotective maneuvers may affect transcription factors regulating chaperones, co-chaperones, and several HSPs. In particular, a number of experimental studies report that increasing chaperones, and especially HSPs, may improve the outcome of I/R injury. Indeed, an important role for HSPs has been described in both the first and second window of protection (Dangi et al., 2015). Moreover, several drugs may be potentially cardioprotective because of their ability to affect the family of heat shock transcription factors and to promote HSP expression within the heart (Willis and Patterson, 2010, 2013; Tarone and Brancaccio, 2014).

Among transcription factors regulating chaperones, the enhancement of heat shock transcription factor 1 (HSF-1), but not the HSF-2 activity has been described after cardiac I/R in post-ischemic rat heart (Nishizawa et al., 1996). Subsequently, it has been observed that HSF-1 induction in I/R is mediated by ROS and ATP levels (Chang et al., 2001; David et al., 2013). Also, X-box binding protein 1 (XBP1), a transcription factor involved in the endoplasmic reticulum chaperone neo-formation regulates the cellular response to ischemia. Indeed, in hypoxic conditions, a dominant-negative form of XBP1 determines an increase in apoptosis in cardiomyocytes (Thuerauf et al., 2006). Moreover, activating transcription factor 6 (ATF6), a transcription factor boosting endoplasmic reticulum chaperone synthesis that is involved in the unfolded protein response, induces enhanced expression of GRP78 and GRP94 chaperones in response to I/R. ATF6 pharmacological blockade impairs heart function and augments the mortality rate after myocardial ischemia (Delisle et al., 2004; Toko et al., 2010).

HSP72/HSP70 (also known as inducible HSP70) has been the focus of many types of research in I/R and cardioprotection fields. Indeed, it has been reported that HSP70 and small HSPs, such as HSP27, induce cardioprotection against irreversible injury associated with I/R (Moghimian et al., 2014). HSP72 seems to increase at 1 week after coronary artery occlusion (Tanonaka et al., 2003). Its expression in rat hearts, induced after a single oral dose of geranylgeranyl acetone, an antiulcer agent, protects against I/R injury (Ooie et al., 2001). The expression of HSP70, instead, is rapidly induced in the ischemic-reperfused heart (Nishizawa et al., 1996). Cardioprotective effects of HSP70 have been reported in isolated adult cardiac myocytes and in transgenic mouse hearts (Knowlton et al., 1991; Heads et al., 1995; Plumier et al., 1995; Lepore et al., 2001; Okubo et al., 2001). HSP72/HSP70 participates to cardioprotection induced by exercise preconditioning, early and late protection, where HSP70 repairs unfolded proteins or may stabilize the function of the endoplasmic reticulum (Yuan et al., 2018). Although several pieces of evidence suggest that brief ischemia triggers the expression of HSP70 (Polla, 1988; Knowlton et al., 1991; Sun et al., 1995) and that HSP70 is actively associated with myocardial protection (Marber et al., 1995; Plumier et al., 1995; Chiu et al., 2003; Guisasola et al., 2006), it has been suggested that the induction of HSP72, as end effectors of protection in ischemic preconditioning, does not occur in the first, but in the second window of protection.

The overexpression of HSP70 seems also to augment the NO^•^ production in response to cytokine stimulation, thus protecting cultured cells from TNFα injury (Latchman, 2001). HSP70 is also present in the exosomes, small vesicles released from cells into the blood. They can transmit signals with activation of protective pathways in cardiomyocytes via toll-like receptor (TLR) 4. The cardioprotective mechanism of exosomes seems mediated by HSP70, which activates a pathway downstream of TLR4, with action on ERK1/2 and p38MAPK and phosphorylation of HSP27 (Vicencio et al., 2015).

**Figure 3:**
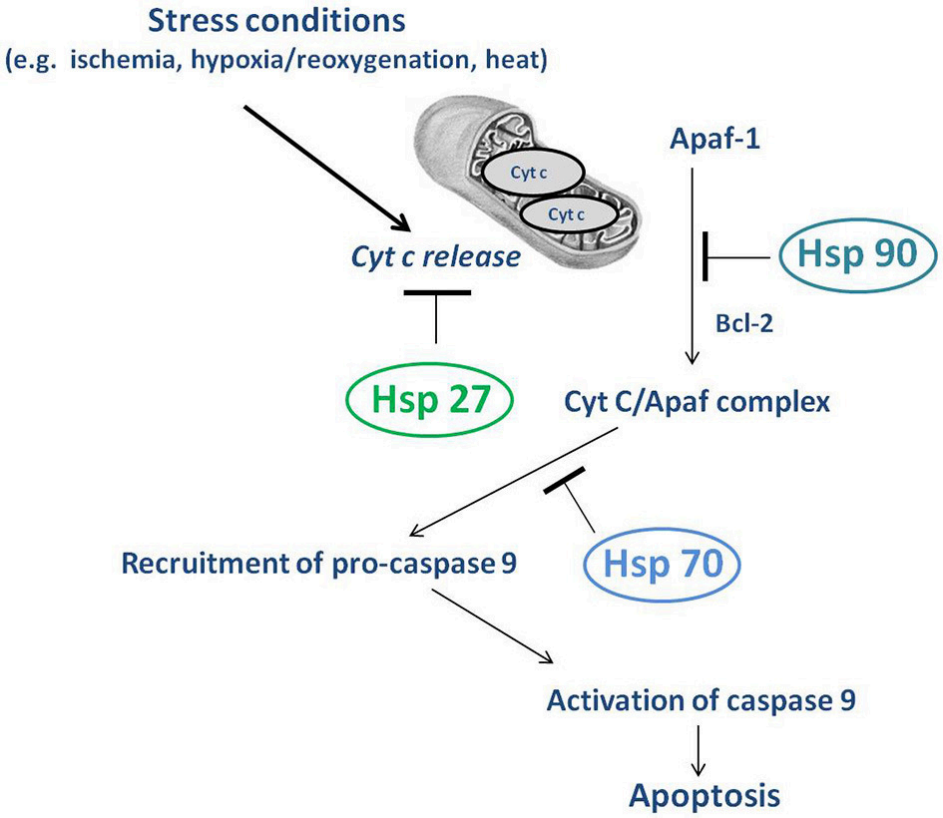
Overview of mitochondrial chaperone proteins.

HSP90 is essential for the integrity and correct function of numerous signaling proteins. The increased expression of HSP90 has been described in the myocardium after I/R (Nishizawa et al., 1996). Indeed, during the ischemic preconditioning, HSP90 is activated by situations of cellular stress and facilitates the mitochondrial importation of cytosolic proteins (Jiao et al., 2008). Intriguingly, HSP90 is involved in the mitochondrial importation of connexin 43, which together with the adenosine triphosphate-sensitive K^+^ channels, is fundamental in cardioprotection from ischemic preconditioning (Rodriguez-Sinovas et al., 2006; Jiao et al., 2008). Furthermore, PostC improves the translocation of PKCε to mitochondria in an HSP90-dependent manner (Zhong et al., 2014). HSP90 is involved in the reduction of apoptosis and cardiomyocyte necrosis, favoring the induction of Bcl-2 anti-apoptotic protein and the inhibition of pro-apoptotic Bax in the mitochondrial fraction (Zhong et al., 2014; Figure 3). Yet, HSP90 binds NOS3 and stimulates its activity (Latchman, 2001) and its overexpression has been reported to reduce I/R lesions via the Akt/NOS3 pathway (Kupatt et al., 2004).

As said above, an HSP90 homolog is the TRAP1/HSP75. It is targeted to mitochondria where is fundamental for mitochondrial integrity and protection from cell death caused by oxidative stress (Montesano Gesualdi et al., 2007). Recent studies have reported the protective action of TRAP1 against I/R-induced mitochondria dysfunction and cell injury (Zhang et al., 2015). In cardiac models both I/R and hypoxia/reoxygenation protocols induced TRAP1 (Xiang et al., 2010; Kim et al., 2012). Its overexpression hinders hypoxia-induced mitochondrial injury and cell death also in isolated rat cardiomyocytes (Williamson et al., 2008).

Melusin is a chaperone protein selectively expressed in cardiac and skeletal muscles, able to act as co-chaperone in the HSP90 machinery. Melusin limits cardiomyocyte death and ameliorates adaptive hypertrophy signaling pathways in response to different stress conditions, including cardiac I/R (Penna et al., 2014b; Tarone and Brancaccio, 2015). The overexpression of Melusin confers resistance to cardiac I/R injury via activation of AKT and ERK kinases and increasing HSP90 expression (Penna et al., 2014b).

HSP60 is a chaperone considered as a good marker for monitoring myocardial damage and heart failure. Intriguingly, high anti-HSP60 antibody levels correlate with high levels of brain natriuretic peptide and with left ventricular end-diastolic dimension, and the HSP60 levels correlate with the extent of cardiac dysfunction (Novo et al., 2011). It has been reported that HSP60 increases few weeks after coronary artery occlusion in rat heart (Tanonaka et al., 2003; Toga et al., 2007; Wang et al., 2010). Also in human ischemic heart disease, HSP60 doubled its expression in comparison to control subjects (Knowlton et al., 1998). HSP60 has different localizations: it is present in the exosomes and in the extra-mitochondrial cytosol of several cells. In the cardiac tissue, the cytosolic HSP60 forms complexes with Bax, Bak, and Bcl-XL, but not with Bcl-2. During hypoxia, HSP60 cellular distribution changes, leaving the cytosol and translocating to the plasma membrane (Gupta and Knowlton, 2005). In ischemic heart disease, instead, HSP60 translocates from the cytoplasm to mitochondria (Sidorik et al., 2005). Increased expression of cardiac HSP60 after 9–12 weeks of coronary artery ligation in rats has been correlated with NF-κB activation (Wang et al., 2010). However, it has been proposed that low doses of lipopolysaccharide could provide a means of reducing myocardial I/R injury by increasing HSP70 with a subsequent inhibition of NF-κB (Yao et al., 2011). This apparent discrepancy on the role of NF-κB may reflect the importance of a sequential involvement of the different chaperones in determining the protective effects in the I/R context.

HSP56/FKBP52 is part of the family of FK506-binding proteins (FKBPs) and behaves as a co-chaperone associated with HSP90 in steroid receptor complexes (Carroll et al., 2011). Recently, it has been reported that in mice the overexpression of HSP56 neither induces myocardial hypertrophy nor protects the intact heart from I/R-injury (Carroll et al., 2011). Although, HSP56 has cardiac action, it seems not protective also when induced by the cardiac-derived peptide cardiotrophin-1, which has cardioprotective properties (Brar et al., 2001).

Small HSPs involved in the response to I/R include HSP20, HSP22, HSP27 and αB-crystallin, which are often increased in response to stress. HSP27 and αB-crystallin increase in response to stress to protect against insults such as I/R (Efthymiou et al., 2004; Arrigo et al., 2007). These two proteins are vital to muscle development and assembly (Brown et al., 2007). Indeed, HSPB2/HSP27 overexpression limits I/R injury in adult cardiomyocytes (Vander Heide, 2002). Elevated levels of HSP27 may also participate to cardioprotection with anti-apoptotic effects. They preserve the integrity of actin cytoskeleton and microtubules and protect the endothelium from ischemia (Mehlen et al., 1996; Latchman, 2001). Indeed, HSP27 behaves as a downstream effector of p38 MAPK during ischemic or β-adrenergic preconditioning or oxytocin protective protocols (Marais et al., 2005; Moghimian et al., 2014).

αB-crystallin has different cellular locations and its phosphorylation is necessary for its activation and translocation to mitochondria and microfilaments (Jin et al., 2008). αB-crystallin seems to bind to the Voltage-Dependent Anion-selective Channel 1 (VDAC1) during hypoxic/redox stresses in neonatal mouse cardiomyocytes (Chis et al., 2012) and to both VDAC1 and ANT during myocardial infarction. αB-crystallin mitochondrial translocation inhibits cytochrome c release into the cytosol. αB-crystallin, by binding to different target molecules, results protective for cardiomyocytes by preserving sarcomeric elasticity, mitochondrial integrity and redox balance (Bullard et al., 2004; Maloyan et al., 2005; Rajasekaran et al., 2007). Indeed, during ischemia, αB-crystallin is phosphorylated and translocated to the contractile cell apparatus where interacts with several cytoskeletal proteins, such as desmin and actin, to maintain protein folding and to prevent aggregation (Bennardini et al., 1992; Djabali et al., 1997; Golenhofen et al., 1998; Wang et al., 2002, 2003). After I/R insult, αB-crystallin translocates to mitochondria (Martindale et al., 2005) where it may exert cardioprotective effects. Indeed, αB-crystallin KO mice show decreased contractile recovery with increased necrosis and apoptosis (Ray et al., 2001; Morrison et al., 2004; Bousette et al., 2010). Yet, cardioprotective post-conditioning induces a preservation of αB-crystallin levels in pigs and αB-crystallin-peptide administration in mice limits infarct area (Cubedo et al., 2016). Recently, it has been reported that subchronic nandrolone administration limits cardiac oxidative stress by inducing the expression of antioxidant proteins, comprising αB-crystallin, thus contributing to amelioration of post-ischemic heart performance (Pergolizzi et al., 2017).

HSP20/HSPB6 is a small HSP located in the cytoplasm that may translocate in part into the nucleus after a heart stress. Induced expression of HSP20/HSPB6 limits apoptosis and infarct size and improves cardiac contractility. HSP20 expression in I/R seems regulated, at least in part, by miR-320 (Ren et al., 2009). Inhibition of HSP20 phosphorylation may exacerbate cardiac I/R damage by suppressing autophagy and increasing other modalities of cell death (Qian et al., 2009; Edwards et al., 2011; Fan and Kranias, 2011). In ischemic conditions, HSP20 is associated to the sarcomeric structure in cardiac and skeletal muscle, as well as in cardiac myoblast cell line, H9C2 (van de Klundert and de Jong, 1999; Verschuure et al., 2002; Golenhofen et al., 2004). In cardiac cells, isoproterenol treatment induced a redistribution of HSP20 to the cytoskeleton and co-localization with actin. HSP20 can be phosphorylated in three phosphorylation sites: serine 16 by PKA/PKG; serine 59 through PKC; and serine 157 via insulin stimulation (Fan et al., 2005). Its phosphorylation at Ser16 may provide cardioprotection against β-agonist-induced apoptosis (Fan et al., 2004). Moreover, HSP20 may interact with the Bcl-2 family and the proapoptotic protein Bax. The anti-apoptotic effect of HSP20 is mediated by PKA pathway, and it prevents the translocation of Bax from the cytosol to the mitochondria, thus limiting cytochrome c release and caspase-3 activation (Fan et al., 2005).

HSPB8/HSP22 is another small HSP whose expression is swiftly induced after ischemia. It is increased also 3-fold in a pig model 1 h after reperfusion following ischemia (Depre et al., 2001) and in the hibernating myocardium in humans and swine (Depre et al., 2004). Indeed HSP22 overexpression limits apoptosis and infarct size (Depre et al., 2006; Sui et al., 2009).

Also, co-chaperones are important in determining the response to I/R stress. DnaJ-like pDJA1 increases 4-fold after reperfusion in a pig model of I/R (Depre et al., 2003). BAG-1, another co-chaperone, protects against I/R induced apoptosis (Salo et al., 1991). CHIP, a co-chaperone/ubiquitin ligase involved in protein quality control, is necessary for optimal cardioprotection after coronary occlusion in mice. Indeed, CHIP KO mouse shows a larger infarct size (Zhang et al., 2005). Ubiquitin *(UB)* is a small molecular weight protein best known for its role in the proteasomal degradation of damaged proteins. Recently, it has been reported that exogenous and prolonged treatment with UB before I/R protocol, reduces infarct size, improves heart function and decreases inflammatory response. Whether this protection occurs with the intervention of CHIP is not clear yet (Fehrenbach et al., 2000).

## Conclusions

Protein misfolding and aggregation are emerging as crucial mechanisms in inducing cardiomyopathy and ischemic damage. The ability of chaperone proteins to inhibit unfolded protein aggregation inducing their degradation and to potentiate beneficial signal transduction pathways in cardiomyocytes is responsible for chaperone-mediated cardioprotection in different pathological conditions. ROS/RNS production in the myocardium causes protein modification and unfolding, inducing mitochondrial dysfunction and cardiomyocyte loss. However, the increase in ROS/RNS levels, besides inducing chaperone expression through the activation of specific transcription factors, likely modify cardiac chaperones directly on amino acid residues and/or formation of disulfide bonds, promoting cell survival and cardiac function. This is a fascinating possibility that would contribute to explain the mechanism of conditioning in heart protection and open the way to new possible therapeutic interventions.

## Author contributions

All authors listed have made a substantial, direct and intellectual contribution to the work, and approved it for publication.

### Conflict of interest statement

The authors declare that the research was conducted in the absence of any commercial or financial relationships that could be construed as a potential conflict of interest.

## References

[B1] Anand P. Stamler J. S. (2012). Enzymatic mechanisms regulating protein S-nitrosylation: implications in health and disease. J. Mol. Med. 90, 233–244. 10.1007/s00109-012-0878-z 22361849 PMC3379879

[B2] Arai M. Imai H. Koumura T. Yoshida M. Emoto K. Umeda M.. (1999). Mitochondrial phospholipid hydroperoxide glutathione peroxidase plays a major role in preventing oxidative injury to cells. J. Biol. Chem. 274, 4924–4933. 10.1074/jbc.274.8.4924 9988735

[B3] Arndt V. Dick N. Tawo R. Dreiseidler M. Wenzel D. Hesse M.. (2010). Chaperone-assisted selective autophagy is essential for muscle maintenance. Curr. Biol. 20, 143–148. 10.1016/j.cub.2009.11.022 20060297

[B4] Arrigo A. P. Simon S. Gibert B. Kretz-Remy C. Nivon M. Czekalla A.. (2007). Hsp27 (HspB1) and alphaB-crystallin (HspB5) as therapeutic targets. FEBS Lett. 581, 3665–3674. 10.1016/j.febslet.2007.04.033 17467701

[B5] Bakthisaran R. Tangirala R. Rao Ch M. (2015). Small heat shock proteins: role in cellular functions and pathology. Biochim. Biophys. Acta 1854, 291–319. 10.1016/j.bbapap.2014.12.019 25556000

[B6] Baumann F. Milisav I. Neupert W. Herrmann J. M. (2000). Ecm10, a novel hsp70 homolog in the mitochondrial matrix of the yeast Saccharomyces cerevisiae. FEBS Lett. 487, 307–312. 10.1016/S0014-5793(00)02364-4 11150530

[B7] Beer S. M. Taylor E. R. Brown S. E. Dahm C. C. Costa N. J. Runswick M. J.. (2004). Glutaredoxin 2 catalyzes the reversible oxidation and glutathionylation of mitochondrial membrane thiol proteins: implications for mitochondrial redox regulation and antioxidant DEFENSE. J. Biol. Chem. 279, 47939–47951. 10.1074/jbc.M408011200 15347644

[B8] Beigi F. Gonzalez D. R. Minhas K. M. Sun Q. A. Foster M. W. Khan S. A.. (2012). Dynamic denitrosylation via S-nitrosoglutathione reductase regulates cardiovascular function. Proc. Natl. Acad. Sci. U.S.A. 109, 4314–4319. 10.1073/pnas.1113319109 22366318 PMC3306718

[B9] Beltran Valls M. R. Dimauro I. Brunelli A. Tranchita E. Ciminelli E. Caserotti P.. (2014). Explosive type of moderate-resistance training induces functional, cardiovascular, and molecular adaptations in the elderly. Age 36, 759–772. 10.1007/s11357-013-9584-1 24136652 PMC4039278

[B10] Benhar M. Forrester M. T. Stamler J. S. (2009). Protein denitrosylation: enzymatic mechanisms and cellular functions. Nat. Rev. Mol. Cell Biol. 10, 721–732. 10.1038/nrm2764 19738628

[B11] Benhar M. Forrester M. T. Hess D. T. Stamler J. S. (2008). Regulated protein denitrosylation by cytosolic and mitochondrial thioredoxins. Science 320, 1050–1054. 10.1126/science.1158265 18497292 PMC2754768

[B12] Bennardini F. Wrzosek A. Chiesi M. (1992). Alpha B-crystallin in cardiac tissue. Association with actin and desmin filaments. Circ. Res. 71, 288–294. 10.1161/01.RES.71.2.288 1628387

[B13] Blamowska M. Neupert W. Hell K. (2012). Biogenesis of the mitochondrial Hsp70 chaperone. J. Cell Biol. 199, 125–135. 10.1083/jcb.201205012 23007651 PMC3461517

[B14] Bousette N. Chugh S. Fong V. Isserlin R. Kim K. H. Volchuk A.. (2010). Constitutively active calcineurin induces cardiac endoplasmic reticulum stress and protects against apoptosis that is mediated by alpha-crystallin-B. Proc. Natl. Acad. Sci. U.S.A. 107, 18481–18486. 10.1073/pnas.1013555107 20937869 PMC2972971

[B15] Brar B. K. Stephanou A. Pennica D. Latchman D. S. (2001). CT-1 mediated cardioprotection against ischaemic re-oxygenation injury is mediated by PI3 kinase, Akt and MEK1/2 pathways. Cytokine 16, 93–96. 10.1006/cyto.2001.0951 11741348

[B16] Brinker A. Pfeifer G. Kerner M. J. Naylor D. J. Hartl F. U. Hayer-Hartl M. (2001). Dual function of protein confinement in chaperonin-assisted protein folding. Cell 107, 223–233. 10.1016/S0092-8674(01)00517-7 11672529

[B17] Brown D. D. Christine K. S. Showell C. Conlon F. L. (2007). Small heat shock protein Hsp27 is required for proper heart tube formation. Genesis 45, 667–678. 10.1002/dvg.20340 17987658 PMC2668208

[B18] Brown G. C. Borutaite V. (2007). Nitric oxide and mitochondrial respiration in the heart. Cardiovasc. Res. 75, 283–290. 10.1016/j.cardiores.2007.03.022 17466959

[B19] Bullard B. Ferguson C. Minajeva A. Leake M. C. Gautel M. Labeit D.. (2004). Association of the chaperone alphaB-crystallin with titin in heart muscle. J. Biol. Chem. 279, 7917–7924. 10.1074/jbc.M307473200 14676215

[B20] Bulteau A. L. Lundberg K. C. Humphries K. M. Sadek H. A. Szweda P. A. Friguet B.. (2001). Oxidative modification and inactivation of the proteasome during coronary occlusion/reperfusion. J. Biol. Chem. 276, 30057–30063. 10.1074/jbc.M100142200 11375979

[B21] Calderwood S. K. Gong J. Murshid A. (2016). Extracellular HSPs: the complicated roles of extracellular HSPs in immunity. Front. Immunol. 7:159. 10.3389/fimmu.2016.00159 27199984 PMC4842758

[B22] Carra S. Seguin S. J. Landry J. (2008). HspB8 and Bag3: a new chaperone complex targeting misfolded proteins to macroautophagy. Autophagy 4, 237–239. 10.4161/auto.5407 18094623

[B23] Carroll C. J. Suleman N. Davidson S. M. Faulkes D. J. Diss J. K. Knight R. (2011). Transgenic overexpression of HSP56 does not result in cardiac hypertrophy nor protect from ischaemia/reperfusion injury. Int. J. Biochem. Cell Biol. 43, 74–79. 10.1016/j.biocel.2010.09.020 20932935

[B24] Chang J. Knowlton A. A. Xu F. Wasser J. S. (2001). Activation of the heat shock response: relationship to energy metabolites. A ^31^P NMR study in rat hearts. Am. J. Physiol. Heart Circ. Physiol. 280, H426–H433. 10.1152/ajpheart.2001.280.1.H426 11123260

[B25] Chen W. Y. Chang F. R. Huang Z. Y. Chen J. H. Wu Y. C. Wu C. C. (2008). Tubocapsenolide A, a novel withanolide, inhibits proliferation and induces apoptosis in MDA-MB-231 cells by thiol oxidation of heat shock proteins. J. Biol. Chem. 283, 17184–17193. 10.1074/jbc.M709447200 18442981

[B26] Cheng L. H. Hung K. F. Lee T. C. Huang C. Y. Chiu W. T. Lo J. F.. (2016). Mitochondrial co-chaperone protein Tid1 is required for energy homeostasis during skeletal myogenesis. Stem Cell Res. Ther. 7:185. 10.1186/s13287-016-0443-8 27927223 PMC5143475

[B27] Chis R. Sharma P. Bousette N. Miyake T. Wilson A. Backx P. H.. (2012). alpha-Crystallin B prevents apoptosis after H_2_O_2_ exposure in mouse neonatal cardiomyocytes. Am. J. Physiol. Heart Circ. Physiol. 303, H967–H978. 10.1152/ajpheart.00040.2012 22904156 PMC3706333

[B28] Chiu J. H. Tsou M. T. Tung H. H. Tai C. H. Tsai S. K. Chih C. L.. (2003). Preconditioned somatothermal stimulation on median nerve territory increases myocardial heat shock protein 70 and protects rat hearts against ischemia-reperfusion injury. J. Thorac. Cardiovasc. Surg. 125, 678–685. 10.1067/mtc.2003.29 12658212

[B29] Chong K. Y. Lai C. C. Lille S. Chang C. Su C. Y. (1998). Stable overexpression of the constitutive form of heat shock protein 70 confers oxidative protection. J. Mol. Cell. Cardiol. 30, 599–608. 10.1006/jmcc.1997.0623 9515035

[B30] Christians E. S. Ishiwata T. Benjamin I. J. (2012). Small heat shock proteins in redox metabolism: implications for cardiovascular diseases. Int. J. Biochem. Cell Biol. 44, 1632–1645. 10.1016/j.biocel.2012.06.006 22710345 PMC3412898

[B31] Christians E. S. Mustafi S. B. Benjamin I. J. (2014). Chaperones and cardiac misfolding protein diseases. Curr. Protein Pept. Sci. 15, 189–204. 10.2174/1389203715666140331111518 24694370

[B32] Conway M. E. Lee C. (2015). The redox switch that regulates molecular chaperones. Biomol. Concepts 6, 269–284. 10.1515/bmc-2015-0015 26352357

[B33] Cubedo J. Vilahur G. Casaní L. Mendieta G. Gómez-Jabalera E. Juan-Babot O.. (2016). Targeting the molecular mechanisms of ischemic damage: protective effects of alpha-crystallin-B. Int. J. Cardiol. 215, 406–416. 10.1016/j.ijcard.2016.04.072 27128573

[B34] Cumming K. T. Raastad T. Holden G. Bastani N. E. Schneeberger D. Paronetto M. P.. (2014). Effects of vitamin C and E supplementation on endogenous antioxidant systems and heat shock proteins in response to endurance training. Physiol. Rep. 2:e12142. 10.14814/phy2.12142 25293598 PMC4254089

[B35] Dangi S. S. Gupta M. Dangi S. K. Chouhan V. S. Maurya V. P. Kumar P.. (2015). Expression of HSPs: an adaptive mechanism during long-term heat stress in goats (Capra hircus). Int. J. Biometeorol. 59, 1095–1106. 10.1007/s00484-014-0922-5 25348887

[B36] D'Autréaux B. Toledano M. B. (2007). ROS as signalling molecules: mechanisms that generate specificity in ROS homeostasis. Nat. Rev. Mol. Cell Biol. 8, 813–824. 10.1038/nrm2256 17848967

[B37] David S. Bucchieri F. Corrao S. Czarnecka A. M. Campanella C. Farina F.. (2013). Hsp10: anatomic distribution, functions, and involvement in human disease. Front. Biosci. 5, 768–778. 10.2741/E657 23277031

[B38] Davies K. J. (2016). Adaptive homeostasis. Mol. Aspects Med. 49, 1–7. 10.1016/j.mam.2016.04.007 27112802 PMC4868097

[B39] Dekker P. J. Pfanner N. (1997). Role of mitochondrial GrpE and phosphate in the ATPase cycle of matrix Hsp70. J. Mol. Biol. 270, 321–327. 10.1006/jmbi.1997.1131 9237899

[B40] Del Monte F. Agnetti G. (2014). Protein post-translational modifications and misfolding: new concepts in heart failure. Proteomics Clin. Appl. 8, 534–542. 10.1002/prca.201400037 24946239 PMC4560347

[B41] Delisle B. P. Anson B. D. Rajamani S. January C. T. (2004). Biology of cardiac arrhythmias: ion channel protein trafficking. Circ. Res. 94, 1418–1428. 10.1161/01.RES.0000128561.28701.ea 15192037

[B42] Depre C. Kim S. J. John A. S. Huang Y. Rimoldi O. E. Pepper J. R.. (2004). Program of cell survival underlying human and experimental hibernating myocardium. Circ. Res. 95, 433–440. 10.1161/01.RES.0000138301.42713.18 15242971

[B43] Depre C. Tomlinson J. E. Kudej R. K. Gaussin V. Thompson E. Kim S. J.. (2001). Gene program for cardiac cell survival induced by transient ischemia in conscious pigs. Proc. Natl. Acad. Sci. U.S.A. 98, 9336–9341. 10.1073/pnas.171297498 11481491 PMC55421

[B44] Depre C. Wang L. Sui X. Qiu H. Hong C. Hedhli N.. (2006). H11 kinase prevents myocardial infarction by preemptive preconditioning of the heart. Circ. Res. 98, 280–288. 10.1161/01.RES.0000201284.45482.e8 16373598

[B45] Depre C. Wang L. Tomlinson J. E. Gaussin V. Abdellatif M. Topper J. N.. (2003). Characterization of pDJA1, a cardiac-specific chaperone found by genomic profiling of the post-ischemic swine heart. Cardiovasc. Res. 58, 126–135. 10.1016/S0008-6363(02)00845-3 12667953

[B46] Dimauro I. Mercatelli N. Caporossi D. (2016). Exercise-induced ROS in heat shock proteins response. Free Radic. Biol. Med. 98, 46–55. 10.1016/j.freeradbiomed.2016.03.028 27021964

[B47] Djabali K. de Nechaud B. Landon F. Portier M. M. (1997). AlphaB-crystallin interacts with intermediate filaments in response to stress. J. Cell Sci. 110(Pt 21), 2759–2769. 9427392 10.1242/jcs.110.21.2759

[B48] Eaton P. Fuller W. Shattock M. J. (2002). S-thiolation of HSP27 regulates its multimeric aggregate size independently of phosphorylation. J. Biol. Chem. 277, 21189–21196. 10.1074/jbc.M200591200 11925435

[B49] Edwards H. V. Cameron R. T. Baillie G. S. (2011). The emerging role of HSP20 as a multifunctional protective agent. Cell. Signal. 23, 1447–1454. 10.1016/j.cellsig.2011.05.009 21616144

[B50] Efthymiou C. A. Mocanu M. M. de Belleroche J. Wells D. J. Latchmann D. S. Yellon D. M. (2004). Heat shock protein 27 protects the heart against myocardial infarction. Basic Res. Cardiol. 99, 392–394. 10.1007/s00395-004-0483-6 15309411

[B51] El Hindy M. Hezwani M. Corry D. Hull J. El Amraoui F. Harris M.. (2014). The branched-chain aminotransferase proteins: novel redox chaperones for protein disulfide isomerase–implications in Alzheimer's disease. Antioxid. Redox Signal. 20, 2497–2513. 10.1089/ars.2012.4869 24094038 PMC4026213

[B52] Eustace B. K. Jay D. G. (2004). Extracellular roles for the molecular chaperone, hsp90. Cell Cycle 3, 1098–1100. 10.4161/cc.3.9.1088 15326368

[B53] Fan G. C. Kranias E. G. (2011). Small heat shock protein 20 (HspB6) in cardiac hypertrophy and failure. J. Mol. Cell. Cardiol. 51, 574–577. 10.1016/j.yjmcc.2010.09.013 20869365 PMC3033453

[B54] Fan G. C. Chu G. Kranias E. G. (2005). Hsp20 and its cardioprotection. Trends Cardiovasc. Med. 15, 138–141. 10.1016/j.tcm.2005.05.004 16099377

[B55] Fan G. C. Chu G. Mitton B. Song Q. Yuan Q. Kranias E. G. (2004). Small heat-shock protein Hsp20 phosphorylation inhibits beta-agonist-induced cardiac apoptosis. Circ. Res. 94, 1474–1482. 10.1161/01.RES.0000129179.66631.00 15105294

[B56] Fehrenbach E. Niess A. M. Schlotz E. Passek F. Dickhuth H. H. Northoff H. (2000). Transcriptional and translational regulation of heat shock proteins in leukocytes of endurance runners. J. Appl. Physiol. 89, 704–710. 10.1152/jappl.2000.89.2.704 10926657

[B57] Fenton W. A. Weissman J. S. Horwich A. L. (1996). Putting a lid on protein folding: structure and function of the co-chaperonin, GroES. Chem. Biol. 3, 157–161. 10.1016/S1074-5521(96)90257-4 8807841

[B58] Ficker E. Dennis A. T. Wang L. Brown A. M. (2003). Role of the cytosolic chaperones Hsp70 and Hsp90 in maturation of the cardiac potassium channel HERG. Circ. Res. 92, e87–e100. 10.1161/01.RES.0000079028.31393.15 12775586

[B59] Fischer C. P. Hiscock N. J. Basu S. Vessby B. Kallner A. Sjöberg L. B.. (2006). Vitamin E isoform-specific inhibition of the exercise-induced heat shock protein 72 expression in humans. J. Appl. Physiol. 100, 1679–1687. 10.1152/japplphysiol.00421.2005 16384840

[B60] Fratelli M. Demol H. Puype M. Casagrande S. Eberini I. Salmona M.. (2002). Identification by redox proteomics of glutathionylated proteins in oxidatively stressed human T lymphocytes. Proc. Natl. Acad. Sci. U.S.A. 99, 3505–3510. 10.1073/pnas.052592699 11904414 PMC122553

[B61] Giles N. M. Watts A. B. Giles G. I. Fry F. H. Littlechild J. A. Jacob C. (2003). Metal and redox modulation of cysteine protein function. Chem. Biol. 10, 677–693. 10.1016/S1074-5521(03)00174-1 12954327

[B62] Golenhofen N. Ness W. Koob R. Htun P. Schaper W. Drenckhahn D. (1998). Ischemia-induced phosphorylation and translocation of stress protein alpha B-crystallin to Z lines of myocardium. Am. J. Physiol. 274(5 Pt 2), H1457–H1464. 9612350 10.1152/ajpheart.1998.274.5.H1457

[B63] Golenhofen N. Perng M. D. Quinlan R. A. Drenckhahn D. (2004). Comparison of the small heat shock proteins alphaB-crystallin, MKBP, HSP25, HSP20, and cvHSP in heart and skeletal muscle. Histochem. Cell Biol. 122, 415–425. 10.1007/s00418-004-0711-z 15480735

[B64] Graumann J. Lilie H. Tang X. Tucker K. A. Hoffmann J. H. Vijayalakshmi J.. (2001). Activation of the redox-regulated molecular chaperone Hsp33–a two-step mechanism. Structure 9, 377–387. 10.1016/S0969-2126(01)00599-8 11377198

[B65] Groenendyk J. Sreenivasaiah P. K. Kim D. H. Agellon L. B. Michalak M. (2010). Biology of endoplasmic reticulum stress in the heart. Circ. Res. 107, 1185–1197. 10.1161/CIRCRESAHA.110.227033 21071716

[B66] Guisasola M. C. Desco Mdel M. Gonzalez F. S. Asensio F. Dulin E. Suarez A.. (2006). Heat shock proteins, end effectors of myocardium ischemic preconditioning? Cell Stress Chaperones 11, 250–258. 10.1379/CSC-181R1.1 17009598 PMC1576469

[B67] Gupta S. Knowlton A. A. (2005). HSP60, Bax, apoptosis and the heart. J. Cell. Mol. Med. 9, 51–58. 10.1111/j.1582-4934.2005.tb00336.x 15784164 PMC6741334

[B68] Guzzo G. Sciacovelli M. Bernardi P. Rasola A. (2014). Inhibition of succinate dehydrogenase by the mitochondrial chaperone TRAP1 has anti-oxidant and anti-apoptotic effects on tumor cells. Oncotarget 5, 11897–11908. 10.18632/oncotarget.2472 25564869 PMC4323003

[B69] Hartl F. U. Bracher A. Hayer-Hartl M. (2011). Molecular chaperones in protein folding and proteostasis. Nature 475, 324–332. 10.1038/nature10317 21776078

[B70] Haslbeck M. Vierling E. (2015). A first line of stress defense: small heat shock proteins and their function in protein homeostasis. J. Mol. Biol. 427, 1537–1548. 10.1016/j.jmb.2015.02.002 25681016 PMC4360138

[B71] Hatahet F. Ruddock L. W. (2009). Protein disulfide isomerase: a critical evaluation of its function in disulfide bond formation. Antioxid. Redox Signal. 11, 2807–2850. 10.1089/ars.2009.2466 19476414

[B72] Haynes C. M. Ron D. (2010). The mitochondrial UPR - protecting organelle protein homeostasis. J. Cell Sci. 123(Pt 22), 3849–3855. 10.1242/jcs.075119 21048161

[B73] Heads R. J. Latchman D. S. Yellon D. M. (1995). Differential stress protein mRNA expression during early ischaemic preconditioning in the rabbit heart and its relationship to adenosine receptor function. J. Mol. Cell. Cardiol. 27, 2133–2148. 10.1016/S0022-2828(95)91299-1 8576930

[B74] Henstridge D. C. Febbraio M. A. Hargreaves M. (2016). Heat shock proteins and exercise adaptations. Our knowledge thus far and the road still ahead. J. Appl. Physiol. 120, 683–691. 10.1152/japplphysiol.00811.2015 26679615

[B75] Herrmann J. M. Stuart R. A. Craig E. A. Neupert W. (1994). Mitochondrial heat shock protein 70, a molecular chaperone for proteins encoded by mitochondrial DNA. J. Cell Biol. 127, 893–902. 10.1083/jcb.127.4.893 7962074 PMC2200046

[B76] Hess D. T. Matsumoto A. Kim S. O. Marshall H. E. Stamler J. S. (2005). Protein S-nitrosylation: purview and parameters. Nat. Rev. Mol. Cell Biol. 6, 150–166. 10.1038/nrm1569 15688001

[B77] Hurd T. R. Costa N. J. Dahm C. C. Beer S. M. Brown S. E. Filipovska A.. (2005a). Glutathionylation of mitochondrial proteins. Antioxid. Redox Signal. 7, 999–1010. 10.1089/ars.2005.7.999 15998254

[B78] Hurd T. R. Filipovska A. Costa N. J. Dahm C. C. Murphy M. P. (2005b). Disulphide formation on mitochondrial protein thiols. Biochem. Soc. Trans. 33(Pt 6), 1390–1393. 10.1042/BST0331390 16246126

[B79] Ilbert M. Horst J. Ahrens S. Winter J. Graf P. C. Lilie H.. (2007). The redox-switch domain of Hsp33 functions as dual stress sensor. Nat. Struct. Mol. Biol. 14, 556–563. 10.1038/nsmb1244 17515905 PMC2782886

[B80] Imlay J. A. (2003). Pathways of oxidative damage. Annu. Rev. Microbiol. 57, 395–418. 10.1146/annurev.micro.57.030502.090938 14527285

[B81] Jang H. H. Lee K. O. Chi Y. H. Jung B. G. Park S. K. Park J. H.. (2004). Two enzymes in one; two yeast peroxiredoxins display oxidative stress-dependent switching from a peroxidase to a molecular chaperone function. Cell 117, 625–635. 10.1016/j.cell.2004.05.002 15163410

[B82] Jiao J. D. Garg V. Yang B. Hu K. (2008). Novel functional role of heat shock protein 90 in ATP-sensitive K+ channel-mediated hypoxic preconditioning. Cardiovasc. Res. 77, 126–133. 10.1093/cvr/cvm028 18006464

[B83] Jin J. K. Whittaker R. Glassy M. S. Barlow S. B. Gottlieb R. A. Glembotski C. C. (2008). Localization of phosphorylated alphaB-crystallin to heart mitochondria during ischemia-reperfusion. Am. J. Physiol. Heart Circ. Physiol. 294, H337–H344. 10.1152/ajpheart.00881.2007 17993600

[B84] Kang B. H. Plescia J. Dohi T. Rosa J. Doxsey S. J. Altieri D. C. (2007). Regulation of tumor cell mitochondrial homeostasis by an organelle-specific Hsp90 chaperone network. Cell 131, 257–270. 10.1016/j.cell.2007.08.028 17956728

[B85] Khassaf M. Child R. B. McArdle A. Brodie D. A. Esanu C. Jackson M. J. (2001). Time course of responses of human skeletal muscle to oxidative stress induced by nondamaging exercise. J. Appl. Physiol. 90, 1031–1035. 10.1152/jappl.2001.90.3.1031 11181616

[B86] Kim H. K. Kang S. W. Jeong S. H. Kim N. Ko J. H. Bang H.. (2012). Identification of potential target genes of cardioprotection against ischemia-reperfusion injury by express sequence tags analysis in rat hearts. J. Cardiol. 60, 98–110. 10.1016/j.jjcc.2012.02.004 22512836

[B87] Knowlton A. A. Brecher P. Apstein C. S. (1991). Rapid expression of heat shock protein in the rabbit after brief cardiac ischemia. J. Clin. Invest. 87, 139–147. 10.1172/JCI114963 1985091 PMC295010

[B88] Knowlton A. A. Kapadia S. Torre-Amione G. Durand J. B. Bies R. Young J.. (1998). Differential expression of heat shock proteins in normal and failing human hearts. J. Mol. Cell. Cardiol. 30, 811–818. 10.1006/jmcc.1998.0646 9602430

[B89] Kriegenburg F. Ellgaard L. Hartmann-Petersen R. (2012). Molecular chaperones in targeting misfolded proteins for ubiquitin-dependent degradation. FEBS J. 279, 532–542. 10.1111/j.1742-4658.2011.08456.x 22177318

[B90] Krzewska J. Langer T. Liberek K. (2001). Mitochondrial Hsp78, a member of the Clp/Hsp100 family in Saccharomyces cerevisiae, cooperates with Hsp70 in protein refolding. FEBS Lett. 489, 92–96. 10.1016/S0014-5793(00)02423-6 11231020

[B91] Kupatt C. Dessy C. Hinkel R. Raake P. Daneau G. Bouzin C.. (2004). Heat shock protein 90 transfection reduces ischemia-reperfusion-induced myocardial dysfunction via reciprocal endothelial NO synthase serine 1177 phosphorylation and threonine 495 dephosphorylation. Arterioscler. Thromb. Vasc. Biol. 24, 1435–1441. 10.1161/01.ATV.0000134300.87476.d1 15178564

[B92] Lacza Z. Pankotai E. Busija D. W. (2009). Mitochondrial nitric oxide synthase: current concepts and controversies. Front. Biosci. 14, 4436–4443. 10.2741/3539 19273361 PMC4570492

[B93] Latchman D. S. (2001). Heat shock proteins and cardiac protection. Cardiovasc. Res. 51, 637–646. 10.1016/S0008-6363(01)00354-6 11530097

[B94] Lau J. K. Pennings G. J. Yong A. Kritharides L. (2017). Cardiac remote ischaemic preconditioning: mechanistic and clinical considerations. Heart Lung Circ. 26, 545–553. 10.1016/j.hlc.2016.11.006 28094122

[B95] Lavery L. A. Partridge J. R. Ramelot T. A. Elnatan D. Kennedy M. A. Agard D. A. (2014). Structural asymmetry in the closed state of mitochondrial Hsp90 (TRAP1) supports a two-step ATP hydrolysis mechanism. Mol. Cell 53, 330–343. 10.1016/j.molcel.2013.12.023 24462206 PMC3947485

[B96] Lepore D. A. Knight K. R. Anderson R. L. Morrison W. A. (2001). Role of priming stresses and Hsp70 in protection from ischemia-reperfusion injury in cardiac and skeletal muscle. Cell Stress Chaperones 6, 93–96. 10.1379/1466-1268(2001)006<0093:ROPSAH>2.0.CO;2 11599579 PMC434395

[B97] Lin K. M. Hollander J. M. Kao V. Y. Lin B. Macpherson L. Dillmann W. H. (2004). Myocyte protection by 10 kD heat shock protein (Hsp10) involves the mobile loop and attenuation of the Ras GTP-ase pathway. FASEB J. 18, 1004–1006. 10.1096/fj.03-0348fje 15059967

[B98] Lind C. Gerdes R. Hamnell Y. Schuppe-Koistinen I. von Löwenhielm H. B. Holmgren A.. (2002). Identification of S-glutathionylated cellular proteins during oxidative stress and constitutive metabolism by affinity purification and proteomic analysis. Arch. Biochem. Biophys. 406, 229–240. 10.1016/S0003-9861(02)00468-X 12361711

[B99] Lutz T. Westermann B. Neupert W. Herrmann J. M. (2001). The mitochondrial proteins Ssq1 and Jac1 are required for the assembly of iron sulfur clusters in mitochondria. J. Mol. Biol. 307, 815–825. 10.1006/jmbi.2001.4527 11273703

[B100] Maloyan A. Sanbe A. Osinska H. Westfall M. Robinson D. Imahashi K.. (2005). Mitochondrial dysfunction and apoptosis underlie the pathogenic process in alpha-B-crystallin desmin-related cardiomyopathy. Circulation 112, 3451–3461. 10.1161/CIRCULATIONAHA.105.572552 16316967 PMC1398051

[B101] Marais E. Genade S. Salie R. Huisamen B. Maritz S. Moolman J. A.. (2005). The temporal relationship between p38 MAPK and HSP27 activation in ischaemic and pharmacological preconditioning. Basic Res. Cardiol. 100, 35–47. 10.1007/s00395-004-0495-7 15526116

[B102] Marber M. S. Mestril R. Chi S. H. Sayen M. R. Yellon D. M. Dillmann W. H. (1995). Overexpression of the rat inducible 70-kD heat stress protein in a transgenic mouse increases the resistance of the heart to ischemic injury. J. Clin. Invest. 95, 1446–1456. 10.1172/JCI117815 7706448 PMC295626

[B103] Martin J. Mayhew M. Langer T. Hartl F. U. (1993). The reaction cycle of GroEL and GroES in chaperonin-assisted protein folding. Nature 366, 228–233. 10.1038/366228a0 7901770

[B104] Martindale J. J. Wall J. A. Martinez-Longoria D. M. Aryal P. Rockman H. A. Guo Y.. (2005). Overexpression of mitogen-activated protein kinase kinase 6 in the heart improves functional recovery from ischemia *in vitro* and protects against myocardial infarction *in vivo*. J. Biol. Chem. 280, 669–676. 10.1074/jbc.M406690200 15492008 PMC3691679

[B105] Martínez-Ruiz A. Araújo I. M. Izquierdo-Álvarez A. Hernansanz-Agustín P. Lamas S. Serrador J. M. (2013). Specificity in S-nitrosylation: a short-range mechanism for NO signaling? Antioxid. Redox Signal. 19, 1220–1235. 10.1089/ars.2012.5066 23157283 PMC3785806

[B106] Martínez-Ruiz A. Villanueva L. González de Orduña C. López-Ferrer D. Higueras M. A. Tarín C.. (2005). S-nitrosylation of Hsp90 promotes the inhibition of its ATPase and endothelial nitric oxide synthase regulatory activities. Proc. Natl. Acad. Sci. U.S.A. 102, 8525–8530. 10.1073/pnas.0407294102 15937123 PMC1150803

[B107] McDonough H. Patterson C. (2003). CHIP: a link between the chaperone and proteasome systems. Cell Stress Chaperones 8, 303–308. 10.1379/1466-1268(2003)008<0303:CALBTC>2.0.CO;2 15115282 PMC514901

[B108] McLendon P. M. Robbins J. (2015). Proteotoxicity and cardiac dysfunction. Circ. Res. 116, 1863–1882. 10.1161/CIRCRESAHA.116.305372 25999425 PMC4443853

[B109] Mehlen P. Schulze-Osthoff K. Arrigo A. P. (1996). Small stress proteins as novel regulators of apoptosis. Heat shock protein 27 blocks Fas/APO-1- and staurosporine-induced cell death. J. Biol. Chem. 271, 16510–16514. 10.1074/jbc.271.28.16510 8663291

[B110] Moghimian M. Faghihi M. Karimian S. M. Imani A. Mobasheri M. B. (2014). Upregulated Hsp27 expression in the cardioprotection induced by acute stress and oxytocin in ischemic reperfused hearts of the rat. Chin. J. Physiol. 57, 329–334. 10.4077/CJP.2014.BAC257 25575521

[B111] Montesano Gesualdi N. Chirico G. Pirozzi G. Costantino E. Landriscina M. Esposito F. (2007). Tumor necrosis factor-associated protein 1 (TRAP-1) protects cells from oxidative stress and apoptosis. Stress 10, 342–350. 10.1080/10253890701314863 17853063

[B112] Morrison L. E. Whittaker R. J. Klepper R. E. Wawrousek E. F. Glembotski C. C. (2004). Roles for alphaB-crystallin and HSPB2 in protecting the myocardium from ischemia-reperfusion-induced damage in a KO mouse model. Am. J. Physiol. Heart Circ. Physiol. 286, H847–H855. 10.1152/ajpheart.00715.2003 14592939

[B113] Morton J. P. Maclaren D. P. Cable N. T. Campbell I. T. Evans L. Kayani A. C.. (2008). Trained men display increased basal heat shock protein content of skeletal muscle. Med. Sci. Sports Exerc. 40, 1255–1262. 10.1249/MSS.0b013e31816a7171 18580405

[B114] Nijtmans L. G. Artal S. M. Grivell L. A. Coates P. J. (2002). The mitochondrial PHB complex: roles in mitochondrial respiratory complex assembly, ageing and degenerative disease. Cell. Mol. Life Sci. 59, 143–155. 10.1007/s00018-002-8411-0 11852914 PMC11337490

[B115] Nishizawa J. Nakai A. Higashi T. Tanabe M. Nomoto S. Matsuda K.. (1996). Reperfusion causes significant activation of heat shock transcription factor 1 in ischemic rat heart. Circulation 94, 2185–2192. 10.1161/01.CIR.94.9.2185 8901670

[B116] Novo G. Cappello F. Rizzo M. Fazio G. Zambuto S. Tortorici E.. (2011). Hsp60 and heme oxygenase-1 (Hsp32) in acute myocardial infarction. Transl. Res. 157, 285–292. 10.1016/j.trsl.2011.01.003 21497776

[B117] Okubo S. Wildner O. Shah M. R. Chelliah J. C. Hess M. L. Kukreja R. C. (2001). Gene transfer of heat-shock protein 70 reduces infarct size *in vivo* after ischemia/reperfusion in the rabbit heart. Circulation 103, 877–881. 10.1161/01.CIR.103.6.877 11171798

[B118] Ooie T. Takahashi N. Saikawa T. Nawata T. Arikawa M. Yamanaka K.. (2001). Single oral dose of geranylgeranylacetone induces heat-shock protein 72 and renders protection against ischemia/reperfusion injury in rat heart. Circulation 104, 1837–1843. 10.1161/hc3901.095771 11591623

[B119] Ostermann J. Horwich A. L. Neupert W. Hartl F. U. (1989). Protein folding in mitochondria requires complex formation with hsp60 and ATP hydrolysis. Nature 341, 125–130. 10.1038/341125a0 2528694

[B120] Parry T. L. Melehani J. H. Ranek M. J. Willis M. S. (2015). Functional amyloid signaling via the inflammasome, necrosome, and signalosome: new therapeutic targets in heart failure. Front. Cardiovasc. Med. 2:25. 10.3389/fcvm.2015.00025 26664897 PMC4671334

[B121] Penna C. Angotti C. Pagliaro P. (2014a). Protein S-nitrosylation in preconditioning and postconditioning. Exp. Biol. Med. 239, 647–662. 10.1177/1535370214522935 24668550

[B122] Penna C. Brancaccio M. Tullio F. Rubinetto C. Perrelli M. G. Angotti C.. (2014b). Overexpression of the muscle-specific protein, melusin, protects from cardiac ischemia/reperfusion injury. Basic Res. Cardiol. 109:418. 10.1007/s00395-014-0418-9 24859929

[B123] Pergolizzi B. Carriero V. Abbadessa G. Penna C. Berchialla P. De Francia S.. (2017). Subchronic nandrolone administration reduces cardiac oxidative markers during restraint stress by modulating protein expression patterns. Mol. Cell. Biochem. 434, 51–60. 10.1007/s11010-017-3036-7 28432552

[B124] Petersen A. C. McKenna M. J. Medved I. Murphy K. T. Brown M. J. Della Gatta P.. (2012). Infusion with the antioxidant N-acetylcysteine attenuates early adaptive responses to exercise in human skeletal muscle. Acta Physiol. 204, 382–392. 10.1111/j.1748-1716.2011.02344.x 21827635

[B125] Petrakis N. Alcock F. Tokatlidis K. (2009). Mitochondrial ATP-independent chaperones. IUBMB Life 61, 909–914. 10.1002/iub.235 19585663

[B126] Plumier J. C. Ross B. M. Currie R. W. Angelidis C. E. Kazlaris H. Kollias G.. (1995). Transgenic mice expressing the human heat shock protein 70 have improved post-ischemic myocardial recovery. J. Clin. Invest. 95, 1854–1860. 10.1172/JCI117865 7706492 PMC295725

[B127] Polla B. S. (1988). A role for heat shock proteins in inflammation? Immunol. Today 9, 134–137. 10.1016/0167-5699(88)91199-1 3256318

[B128] Prip-Buus C. Westerman B. Schmitt M. Langer T. Neupert W. Schwarz E. (1996). Role of the mitochondrial DnaJ homologue, Mdj1p, in the prevention of heat-induced protein aggregation. FEBS Lett. 380, 142–146. 10.1016/0014-5793(96)00049-X 8603724

[B129] Qian J. Ren X. Wang X. Zhang P. Jones W. K. Molkentin J. D.. (2009). Blockade of Hsp20 phosphorylation exacerbates cardiac ischemia/reperfusion injury by suppressed autophagy and increased cell death. Circ. Res. 105, 1223–1231. 10.1161/CIRCRESAHA.109.200378 19850943 PMC2799045

[B130] Radi R. Turrens J. F. Chang L. Y. Bush K. M. Crapo J. D. Freeman B. A. (1991). Detection of catalase in rat heart mitochondria. J. Biol. Chem. 266, 22028–22034. 1657986

[B131] Rajasekaran N. S. Connell P. Christians E. S. Yan L. J. Taylor R. P. Orosz A.. (2007). Human alpha B-crystallin mutation causes oxido-reductive stress and protein aggregation cardiomyopathy in mice. Cell 130, 427–439. 10.1016/j.cell.2007.06.044 17693254 PMC2962423

[B132] Ray P. S. Martin J. L. Swanson E. A. Otani H. Dillmann W. H. Das D. K. (2001). Transgene overexpression of alphaB crystallin confers simultaneous protection against cardiomyocyte apoptosis and necrosis during myocardial ischemia and reperfusion. FASEB J. 15, 393–402. 10.1096/fj.00-0199com 11156955

[B133] Ren X. P. Wu J. Wang X. Sartor M. A. Jones K. Qian J.. (2009). MicroRNA-320 is involved in the regulation of cardiac ischemia/reperfusion injury by targeting heat-shock protein 20. Circulation 119, 2357–2366. 10.1161/CIRCULATIONAHA.108.814145 19380620 PMC2746735

[B134] Rhee S. G. (2006). Cell signaling. H_2_O_2_, a necessary evil for cell signaling. Science 312, 1882–1883. 10.1126/science.1130481 16809515

[B135] Rodriguez-Sinovas A. Boengler K. Cabestrero A. Gres P. Morente M. Ruiz-Meana M.. (2006). Translocation of connexin 43 to the inner mitochondrial membrane of cardiomyocytes through the heat shock protein 90-dependent TOM pathway and its importance for cardioprotection. Circ. Res. 99, 93–101. 10.1161/01.RES.0000230315.56904.de 16741159

[B136] Salo D. C. Donovan C. M. Davies K. J. (1991). HSP70 and other possible heat shock or oxidative stress proteins are induced in skeletal muscle, heart, and liver during exercise. Free Radic. Biol. Med. 11, 239–246. 10.1016/0891-5849(91)90119-N 1937141

[B137] Schilke B. Forster J. Davis J. James P. Walter W. Laloraya S.. (1996). The cold sensitivity of a mutant of Saccharomyces cerevisiae lacking a mitochondrial heat shock protein 70 is suppressed by loss of mitochondrial DNA. J. Cell Biol. 134, 603–613. 10.1083/jcb.134.3.603 8707841 PMC2120932

[B138] Schilke B. Voisine C. Beinert H. Craig E. (1999). Evidence for a conserved system for iron metabolism in the mitochondria of *Saccharomyces cerevisiae*. Proc. Natl. Acad. Sci. U.S.A. 96, 10206–10211. 10.1073/pnas.96.18.10206 10468587 PMC17867

[B139] Schmidt S. Strub A. Röttgers K. Zufall N. Voos W. (2001). The two mitochondrial heat shock proteins 70, Ssc1 and Ssq1, compete for the cochaperone Mge1. J. Mol. Biol. 313, 13–26. 10.1006/jmbi.2001.5013 11601843

[B140] Schneider H. C. Westermann B. Neupert W. Brunner M. (1996). The nucleotide exchange factor MGE exerts a key function in the ATP-dependent cycle of mt-Hsp70-Tim44 interaction driving mitochondrial protein import. EMBO J. 15, 5796–5803. 8918457 PMC452327

[B141] Schwartz M. P. Huang S. Matouschek A. (1999). The structure of precursor proteins during import into mitochondria. J. Biol. Chem. 274, 12759–12764. 10.1074/jbc.274.18.12759 10212260

[B142] Schwarzer C. Barnikol-Watanabe S. Thinnes F. P. Hilschmann N. (2002). Voltage-dependent anion-selective channel (VDAC) interacts with the dynein light chain Tctex1 and the heat-shock protein PBP74. Int. J. Biochem. Cell Biol. 34, 1059–1070. 10.1016/S1357-2725(02)00026-2 12009301

[B143] Sciacovelli M. Guzzo G. Morello V. Frezza C. Zheng L. Nannini N.. (2013). The mitochondrial chaperone TRAP1 promotes neoplastic growth by inhibiting succinate dehydrogenase. Cell Metab. 17, 988–999. 10.1016/j.cmet.2013.04.019 23747254 PMC3677096

[B144] Scroggins B. T. Neckers L. (2007). Post-translational modification of heat-shock protein 90: impact on chaperone function. Expert Opin. Drug Discov. 2, 1403–1414. 10.1517/17460441.2.10.1403 23484535

[B145] Sengupta R. Ryter S. W. Zuckerbraun B. S. Tzeng E. Billiar T. R. Stoyanovsky D. A. (2007). Thioredoxin catalyzes the denitrosation of low-molecular mass and protein S-nitrosothiols. Biochemistry 46, 8472–8483. 10.1021/bi700449x 17580965

[B146] Shan Y. X. Liu T. J. Su H. F. Samsamshariat A. Mestril R. Wang P. H. (2003). Hsp10 and Hsp60 modulate Bcl-2 family and mitochondria apoptosis signaling induced by doxorubicin in cardiac muscle cells. J. Mol. Cell. Cardiol. 35, 1135–1143. 10.1016/S0022-2828(03)00229-3 12967636

[B147] Sidorik L. Kyyamova R. Bobyk V. Kapustian L. Rozhko O. Vigontina O.. (2005). Molecular chaperone, HSP60, and cytochrome P450 2E1 co-expression in dilated cardiomyopathy. Cell Biol. Int. 29, 51–55. 10.1016/j.cellbi.2004.11.011 15763499

[B148] Simar D. Malatesta D. Mas E. Delage M. Caillaud C. (2012). Effect of an 8-weeks aerobic training program in elderly on oxidative stress and HSP72 expression in leukocytes during antioxidant supplementation. J. Nutr. Health Aging 16, 155–161. 10.1007/s12603-011-0106-5 22323351 PMC12878004

[B149] Sorge M. Brancaccio M. (2016). Melusin promotes a protective signal transduction cascade in stressed hearts. Front. Mol. Biosci. 3:53. 10.3389/fmolb.2016.00053 27672636 PMC5018970

[B150] Steglich G. Neupert W. Langer T. (1999). Prohibitins regulate membrane protein degradation by the m-AAA protease in mitochondria. Mol. Cell. Biol. 19, 3435–3442. 10.1128/MCB.19.5.3435 10207067 PMC84136

[B151] Sui X. Li D. Qiu H. Gaussin V. Depre C. (2009). Activation of the bone morphogenetic protein receptor by H11kinase/Hsp22 promotes cardiac cell growth and survival. Circ. Res. 104, 887–895. 10.1161/CIRCRESAHA.108.192328 19246680

[B152] Sun J. Z. Tang X. L. Knowlton A. A. Park S. W. Qiu Y. Bolli R. (1995). Late preconditioning against myocardial stunning. An endogenous protective mechanism that confers resistance to postischemic dysfunction 24 h after brief ischemia in conscious pigs. J. Clin. Invest. 95, 388–403. 10.1172/JCI117667 7814639 PMC295442

[B153] Taipale M. Jarosz D. F. Lindquist S. (2010). HSP90 at the hub of protein homeostasis: emerging mechanistic insights. Nat. Rev. Mol. Cell Biol. 11, 515–528. 10.1038/nrm2918 20531426

[B154] Tannous P. Zhu H. Nemchenko A. Berry J. M. Johnstone J. L. Shelton J. M.. (2008). Intracellular protein aggregation is a proximal trigger of cardiomyocyte autophagy. Circulation 117, 3070–3078. 10.1161/CIRCULATIONAHA.107.763870 18541737 PMC2601596

[B155] Tanonaka K. Toga W. Yoshida H. Takeo S. (2003). Myocardial heat shock protein changes in the failing heart following coronary artery ligation. Heart Lung Circ. 12, 60–65. 10.1046/j.1444-2892.2003.00139.x 16352108

[B156] Tarone G. Brancaccio M. (2014). Keep your heart in shape: molecular chaperone networks for treating heart disease. Cardiovasc. Res. 102, 346–361. 10.1093/cvr/cvu049 24585203

[B157] Tarone G. Brancaccio M. (2015). The muscle-specific chaperone protein melusin is a potent cardioprotective agent. Basic Res. Cardiol. 110:10. 10.1007/s00395-015-0466-9 25653116

[B158] Tarone G. Lembo G. (2003). Molecular interplay between mechanical and humoral signalling in cardiac hypertrophy. Trends Mol. Med. 9, 376–382. 10.1016/S1471-4914(03)00164-3 13129703

[B159] Thuerauf D. J. Marcinko M. Gude N. Rubio M. Sussman M. A. Glembotski C. C. (2006). Activation of the unfolded protein response in infarcted mouse heart and hypoxic cultured cardiac myocytes. Circ. Res. 99, 275–282. 10.1161/01.RES.0000233317.70421.03 16794188

[B160] Tian Z. Zheng H. Li J. Li Y. Su H. Wang X. (2012). Genetically induced moderate inhibition of the proteasome in cardiomyocytes exacerbates myocardial ischemia-reperfusion injury in mice. Circ. Res. 111, 532–542. 10.1161/CIRCRESAHA.112.270983 22740087 PMC3426260

[B161] Tocchetti C. G. Stanley B. A. Murray C. I. Sivakumaran V. Donzelli S. Mancardi D. (2011). Playing with cardiac “redox switches”: the “HNO way” to modulate cardiac function. Antioxid. Redox Signal. 14, 1687–1698. 10.1089/ars.2010.3859 21235349 PMC3066693

[B162] Toga W. Tanonaka K. Takeo S. (2007). Changes in Hsp60 level of the failing heart following acute myocardial infarction and the effect of long-term treatment with trandolapril. Biol. Pharm. Bull. 30, 105–110. 10.1248/bpb.30.105 17202668

[B163] Toko H. Takahashi H. Kayama Y. Okada S. Minamino T. Terasaki F.. (2010). ATF6 is important under both pathological and physiological states in the heart. J. Mol. Cell. Cardiol. 49, 113–120. 10.1016/j.yjmcc.2010.03.020 20380836

[B164] Tullio F. Angotti C. Perrelli M. G. Penna C. Pagliaro P. (2013). Redox balance and cardioprotection. Basic Res. Cardiol. 108:392. 10.1007/s00395-013-0392-7 24158692

[B165] Turrens J. F. (2003). Mitochondrial formation of reactive oxygen species. J. Physiol. 552(Pt 2), 335–344. 10.1113/jphysiol.2003.049478 14561818 PMC2343396

[B166] van de Klundert F. A. de Jong W. W. (1999). The small heat shock proteins Hsp20 and alphaB-crystallin in cultured cardiac myocytes: differences in cellular localization and solubilization after heat stress. Eur. J. Cell Biol. 78, 567–572. 10.1016/S0171-9335(99)80022-3 10494863

[B167] van Dyck L. Dembowski M. Neupert W. Langer T. (1998). Mcx1p, a ClpX homologue in mitochondria of *Saccharomyces cerevisiae*. FEBS Lett. 438, 250–254. 10.1016/S0014-5793(98)01310-6 9827555

[B168] Vander Heide R. S. (2002). Increased expression of HSP27 protects canine myocytes from simulated ischemia-reperfusion injury. Am. J. Physiol. Heart Circ. Physiol. 282, H935–H941. 10.1152/ajpheart.00660.2001 11834489

[B169] Verschuure P. Croes Y. van den IJssel P. R. Quinlan R. A. de Jong W. W. Boelens W. C. (2002). Translocation of small heat shock proteins to the actin cytoskeleton upon proteasomal inhibition. J. Mol. Cell. Cardiol. 34, 117–128. 10.1006/jmcc.2001.1493 11851352

[B170] Vicart P. Caron A. Guicheney P. Li Z. Prévost M. C. Faure A.. (1998). A missense mutation in the alphaB-crystallin chaperone gene causes a desmin-related myopathy. Nat. Genet. 20, 92–95. 10.1038/1765 9731540

[B171] Vicencio J. M. Yellon D. M. Sivaraman V. Das D. Boi-Doku C. Arjun S.. (2015). Plasma exosomes protect the myocardium from ischemia-reperfusion injury. J. Am. Coll. Cardiol. 65, 1525–1536. 10.1016/j.jacc.2015.02.026 25881934

[B172] Vogt M. Puntschart A. Geiser J. Zuleger C. Billeter R. Hoppeler H. (2001). Molecular adaptations in human skeletal muscle to endurance training under simulated hypoxic conditions. J. Appl. Physiol. 91, 173–182. 10.1152/jappl.2001.91.1.173 11408428

[B173] Vos M. J. Zijlstra M. P. Carra S. Sibon O. C. Kampinga H. H. (2011). Small heat shock proteins, protein degradation and protein aggregation diseases. Autophagy 7, 101–103. 10.4161/auto.7.1.13935 21045566

[B174] Voth W. Jakob U. (2017). Stress-activated chaperones: a first line of defense. Trends Biochem. Sci. 42, 899–913. 10.1016/j.tibs.2017.08.006 28893460 PMC5659914

[B175] Wachter C. Schatz G. Glick B. S. (1994). Protein import into mitochondria: the requirement for external ATP is precursor-specific whereas intramitochondrial ATP is universally needed for translocation into the matrix. Mol. Biol. Cell 5, 465–474. 10.1091/mbc.5.4.465 7914441 PMC301055

[B176] Wadhwa R. Taira K. Kaul S. C. (2002). An Hsp70 family chaperone, mortalin/mthsp70/PBP74/Grp75: what, when, and where? Cell Stress Chaperones 7, 309–316. 10.1379/1466-1268(2002)007<0309:AHFCMM>2.0.CO;2 12482206 PMC514830

[B177] Wadhwa R. Takano S. Robert M. Yoshida A. Nomura H. Reddel R. R.. (1998). Inactivation of tumor suppressor p53 by mot-2, a hsp70 family member. J. Biol. Chem. 273, 29586–29591. 10.1074/jbc.273.45.29586 9792667

[B178] Wadhwa R. Yaguchi T. Hasan M. K. Taira K. Kaul S. C. (2003). Mortalin-MPD (mevalonate pyrophosphate decarboxylase) interactions and their role in control of cellular proliferation. Biochem. Biophys. Res. Commun. 302, 735–742. 10.1016/S0006-291X(03)00226-2 12646231

[B179] Wang C. Yu J. Huo L. Wang L. Feng W. Wang C. C. (2012). Human protein-disulfide isomerase is a redox-regulated chaperone activated by oxidation of domain a'. J. Biol. Chem. 287, 1139–1149. 10.1074/jbc.M111.303149 22090031 PMC3256865

[B180] Wang L. Wang X. Wang C. C. (2015). Protein disulfide-isomerase, a folding catalyst and a redox-regulated chaperone. Free Radic. Biol. Med. 83, 305–313. 10.1016/j.freeradbiomed.2015.02.007 25697778

[B181] Wang X. Klevitsky R. Huang W. Glasford J. Li F. Robbins J. (2003). AlphaB-crystallin modulates protein aggregation of abnormal desmin. Circ. Res. 93, 998–1005. 10.1161/01.RES.0000102401.77712.ED 14576194

[B182] Wang X. Osinska H. Gerdes A. M. Robbins J. (2002). Desmin filaments and cardiac disease: establishing causality. J. Card. Fail. 8, S287–S292. 10.1054/jcaf.2002.129279 12555134

[B183] Wang Y. Chen L. Hagiwara N. Knowlton A. A. (2010). Regulation of heat shock protein 60 and 72 expression in the failing heart. J. Mol. Cell. Cardiol. 48, 360–366. 10.1016/j.yjmcc.2009.11.009 19945465 PMC2814075

[B184] Williamson C. L. Dabkowski E. R. Dillmann W. H. Hollander J. M. (2008). Mitochondria protection from hypoxia/reoxygenation injury with mitochondria heat shock protein 70 overexpression. Am. J. Physiol. Heart Circ. Physiol. 294, H249–H256. 10.1152/ajpheart.00775.2007 17982016

[B185] Willis M. S. Patterson C. (2010). Hold me tight: role of the heat shock protein family of chaperones in cardiac disease. Circulation 122, 1740–1751. 10.1161/CIRCULATIONAHA.110.942250 20975010 PMC2976481

[B186] Willis M. S. Patterson C. (2013). Proteotoxicity and cardiac dysfunction–Alzheimer's disease of the heart? N. Engl. J. Med. 368, 455–464. 10.1056/NEJMra1106180 23363499

[B187] Wink D. A. Miranda K. M. Katori T. Mancardi D. Thomas D. D. Ridnour L.. (2003). Orthogonal properties of the redox siblings nitroxyl and nitric oxide in the cardiovascular system: a novel redox paradigm. Am. J. Physiol. Heart Circ. Physiol. 285, H2264–H2276. 10.1152/ajpheart.00531.2003 12855429

[B188] Winter J. Ilbert M. Graf P. C. Ozcelik D. Jakob U. (2008). Bleach activates a redox-regulated chaperone by oxidative protein unfolding. Cell 135, 691–701. 10.1016/j.cell.2008.09.024 19013278 PMC2606091

[B189] Xiang F. Huang Y. S. Shi X. H. Zhang Q. (2010). Mitochondrial chaperone tumour necrosis factor receptor-associated protein 1 protects cardiomyocytes from hypoxic injury by regulating mitochondrial permeability transition pore opening. FEBS J. 277, 1929–1938. 10.1111/j.1742-4658.2010.07615.x 20236315

[B190] Xu Z. Horwich A. L. Sigler P. B. (1997). The crystal structure of the asymmetric GroEL-GroES-(ADP)7 chaperonin complex. Nature 388, 741–750. 10.1038/41944 9285585

[B191] Yao Y. W. Zhang G. H. Zhang Y. Y. Li W. D. Wang C. H. Yin C. Y.. (2011). Lipopolysaccharide pretreatment protects against ischemia/reperfusion injury via increase of HSP70 and inhibition of NF-kappaB. Cell Stress Chaperones 16, 287–296. 10.1007/s12192-010-0242-6 21080136 PMC3077230

[B192] Yoshida S. Tsutsumi S. Muhlebach G. Sourbier C. Lee M. J. Lee S.. (2013). Molecular chaperone TRAP1 regulates a metabolic switch between mitochondrial respiration and aerobic glycolysis. Proc. Natl. Acad. Sci. U.S.A. 110, E1604–E1612. 10.1073/pnas.1220659110 23564345 PMC3637790

[B193] Yuan Y. Pan S. S. Shen Y. J. (2018). Cardioprotection of exercise preconditioning involving heat shock protein 70 and concurrent autophagy: a potential chaperone-assisted selective macroautophagy effect. J. Physiol. Sci. 68, 55–67. 10.1007/s12576-016-0507-7 27928720 PMC10717675

[B194] Zhang C. Xu Z. He X. R. Michael L. H. Patterson C. (2005). CHIP, a cochaperone/ubiquitin ligase that regulates protein quality control, is required for maximal cardioprotection after myocardial infarction in mice. Am. J. Physiol. Heart Circ. Physiol. 288, H2836–H2842. 10.1152/ajpheart.01122.2004 15665051

[B195] Zhang P. Lu Y. Yu D. Zhang D. Hu W. (2015). TRAP1 Provides protection against myocardial ischemia-reperfusion injury by ameliorating mitochondrial dysfunction. Cell. Physiol. Biochem. 36, 2072–2082. 10.1159/000430174 26202366

[B196] Zhong G. Q. Tu R. H. Zeng Z. Y. Li Q. J. He Y. Li S.. (2014). Novel functional role of heat shock protein 90 in protein kinase C-mediated ischemic postconditioning. J. Surg. Res. 189, 198–206. 10.1016/j.jss.2014.01.038 24742623

[B197] Ziemann E. Zembroñ-Lacny A. Kasperska A. Antosiewicz J. Grzywacz T. Garsztka T.. (2013). Exercise training-induced changes in inflammatory mediators and heat shock proteins in young tennis players. J. Sports Sci. Med. 12, 282–289. 24149807 PMC3761842

